# Japanese Clinical Practice Guidelines for Vascular Anomalies 2017

**DOI:** 10.1111/1346-8138.15189

**Published:** 2020-03-22

**Authors:** Hidefumi Mimura, Sadanori Akita, Akihiro Fujino, Masatoshi Jinnin, Mine Ozaki, Keigo Osuga, Hiroki Nakaoka, Eiichi Morii, Akira Kuramochi, Yoko Aoki, Yasunori Arai, Noriko Aramaki, Masanori Inoue, Yuki Iwashina, Tadashi Iwanaka, Shigeru Ueno, Akihiro Umezawa, Michio Ozeki, Junko Ochi, Yoshiaki Kinoshita, Masakazu Kurita, Shien Seike, Nobuyuki Takakura, Masataka Takahashi, Takao Tachibana, Kumiko Chuman, Shuji Nagata, Mitsunaga Narushima, Yasunari Niimi, Shunsuke Nosaka, Taiki Nozaki, Kazuki Hashimoto, Ayato Hayashi, Satoshi Hirakawa, Atsuko Fujikawa, Yumiko Hori, Kentaro Matsuoka, Hideki Mori, Yuki Yamamoto, Shunsuke Yuzuriha, Naoaki Rikihisa, Shoji Watanabe, Shinichi Watanabe, Tatsuo Kuroda, Shunsuke Sugawara, Kosuke Ishikawa, Satoru Sasaki

**Affiliations:** ^1^ Department of Radiology St. Marianna University School of Medicine Kawasaki Japan; ^2^ Department of Plastic Surgery Wound Repair and Regeneration Fukuoka University School of Medicine Fukuoka Japan; ^3^ Division of Surgery National Center for Child Health and Development Tokyo Japan; ^4^ Department of Dermatology Wakayama Medical University Wakayama Japan; ^5^ Department of Plastic, Reconstructive and Aesthetic Surgery Kyorin University School of Medicine Mitaka Japan; ^6^ Department of Diagnostic and Interventional Radiology Osaka University Graduate School of Medicine Suita Japan; ^7^ Department of Plastic Surgery Ehime University Hospital Toon Japan; ^8^ Department of Pathology Osaka University Graduate School of Medicine Suita Japan; ^9^ Department of Dermatology Saitama Medical University Iruma‐gun Japan; ^10^ Department of Medical Genetics Tohoku University School of Medicine Sendai Japan; ^11^ Department of Plastic and Reconstructive Surgery Keio University School of Medicine Tokyo Japan; ^12^ Department of Radiology Keio University School of Medicine Tokyo Japan; ^13^ Department of Pediatric Surgery The University of Tokyo Hospital Tokyo Japan; ^14^ Department of Pediatric Surgery Tokai University School of Medicine Isehara Japan; ^15^ Department of Reproductive Biology Center for Regenerative Medicine National Center for Child Health and Development Tokyo Japan; ^16^ Department of Pediatrics Gifu University Graduate School of Medicine Gifu Japan; ^17^ Department of Diagnostic Radiology Tohoku University Graduate School of Medicine Sendai Japan; ^18^ Department of Pediatric Surgery Niigata University Graduate School of Medical and Dental Sciences Niigata Japan; ^19^ Department of Plastic and Reconstructive Surgery The University of Tokyo Hospital Tokyo Japan; ^20^ Department of Plastic Surgery Osaka University Graduate School of Medicine Suita Japan; ^21^ Department of Signal Transduction Research Institute for Microbial Diseases Osaka University Suita Japan; ^22^ Department of Dermatology Osaka Red Cross Hospital Osaka Japan; ^23^ Department of Dermatology Kanto Central Hospital Tokyo Japan; ^24^ Department of Radiology Kurume University School of Medicine Kurume Japan; ^25^ Department of Plastic and Reconstructive Surgery Graduate School of Medicine Mie University Tsu Japan; ^26^ Department of Neuroendovascular Therapy St. Luke's International Hospital Tokyo Japan; ^27^ Division of Radiology National Center for Child Health and Development Tokyo Japan; ^28^ Department of Radiology St. Luke's International Hospital Tokyo Japan; ^29^ Department of Plastic and Reconstructive Surgery Juntendo University Urayasu Hospital Urayasu Japan; ^30^ Department of Dermatology Hamamatsu University School of Medicine Hamamatsu Japan; ^31^ Department of Pathology Dokkyo Medical University Saitama Medical Center Koshigaya Japan; ^32^ Department of Plastic and Reconstructive Surgery Shinshu University School of Medicine Matsumoto Japan; ^33^ Department of Plastic and Reconstructive Surgery Oyumino Central Hospital Chiba Japan; ^34^ Department of Plastic and Reconstructive Surgery Saitama Children's Medical Center Saitama Japan; ^35^ Department of Dermatology Teikyo University School of Medicine Tokyo Japan; ^36^ Department of Pediatric Surgery Keio University School of Medicine Tokyo Japan; ^37^ Department of Diagnostic Radiology National Cancer Center Hospital Tokyo Japan; ^38^ Department of Plastic and Reconstructive Surgery Faculty of Medicine and Graduate School of Medicine Hokkaido University Sapporo Japan; ^39^ Department of Plastic and Reconstructive Surgery Center for Vascular Anomalies Tonan Hospital Sapporo Japan

**Keywords:** clinical practice, guidelines, hemangioma, vascular anomalies, vascular malformation

## Abstract

The objective was to prepare guidelines to perform the current optimum treatment by organizing effective and efficient treatments of hemangiomas and vascular malformations, confirming the safety and systematizing treatment, employing evidence‐based medicine techniques and aimed at improvement of the outcomes. Clinical questions (CQ) were decided based on the important clinical issues. For document retrieval, key words for published work searches were set for each CQ, and work published from 1980 to the end of September 2014 was searched in PubMed, Cochrane Library and Japana Centra Revuo Medicina databases. The strengths of evidence and recommendations acquired by systematic reviews were determined following the Medical Information Network Distribution System technique. A total of 33 CQ were used to compile recommendations and the subjects included efficacy of resection, sclerotherapy/embolization, drug therapy, laser therapy, radiotherapy and other conservative treatment, differences in appropriate treatment due to the location of lesions and among symptoms, appropriate timing of treatment and tests, and pathological diagnosis deciding the diagnosis. Thus, the Japanese Clinical Practice Guidelines for Vascular Anomalies 2017 have been prepared as the evidence‐based guidelines for the management of vascular anomalies.

## INTRODUCTION

The etiology of vascular anomalies on the body surface and in soft tissue are mostly unclear and no fundamental treatment methods have been established. Many patients visit many medical institutions seeking an expert, being a disadvantage in treatment. Hemangiomas and vascular malformations are frequently termed “hemangioma”, but these are different diseases in the classification proposed by the International Society for Study of Vascular Anomalies (ISSVA),^1^, ^2^ and this classification has been internationally standardized.

The *Clinical Practice Guidelines for Vascular Anomalies 2013* (1st edition)^3^ target general practitioners and the general public, and were prepared aiming at organizing effective and efficient treatments for hemangiomas/vascular malformations, confirming the safety and systematizing treatment using evidence‐based medicine (EBM) techniques. The organization responsible for preparation was the Health, Labor and Welfare Sciences Research Grants (Research on Measures for Intractable Diseases), Research Committee for “Intractable Vascular Anomalies”, and the main committee members were selected from academic societies of plastic surgery and radiology mainly treating hemangiomas and vascular malformations: the Japanese Society of Plastic and Reconstructive Surgery and Japanese Society of Interventional Radiology, and the guidelines were prepared by them.

The *Clinical Practice Guidelines for Vascular Anomalies 2017* were prepared as a revised edition of the *Clinical Practice Guidelines for Vascular Anomalies 2013*. The organization responsible for preparation was the Health, Labor and Welfare Sciences Research Grants (Research on Policy Planning and Evaluation for Rare and Intractable Diseases), Research Committee for Intractable Vascular Anomalies, and the differences from the previous guidelines are setting the objective of summarizing opinions from related academic societies by inviting many committee members from dermatologists, pediatric surgeons, pediatricians, radiologists (diagnostic radiology), and basic researchers including the pathology, molecular biology and epidemiology fields, in addition to plastic surgeons and radiologists (interventional radiology). Because the guidelines were prepared following the reformed *Minds Handbook for Clinical Practice Guideline Development 2014* 4 and *Minds Manual for Clinical Practice Guideline Development Ver. 1.0–2.0*,^5^, ^6^ it was fully revised.

The original text of the guidelines (Japanese version) is comprised of reviews and clinical questions (CQ), but only CQ are presented in this report.

## PURPOSE OF THE GUIDELINES

The objective was to prepare guidelines to perform the current optimum treatment by organizing effective and efficient treatments of hemangiomas and vascular malformations, confirming the safety and systematizing treatment, employing the EBM techniques and aimed at improvement of the following outcomes: pain, swelling, esthetic impairment and functional disorder.

## METHODS

### Organization

For the Guidelines Executive Committee members, representatives of the plastic surgery, dermatology, radiology, pediatric surgery and basic science fields were selected. The guidelines preparation group and systematic review (SR) team for preparation of CQ and recommendations were comprised of four groups: (i) groups in charge of arteriovenous malformation (AVM), venous malformation (VM), combined type and syndrome; (ii) in charge of capillary malformation (CM) and infantile hemangioma; (iii) in charge of the lymphatic malformation (lymphangioma) (LM); and (iv) in charge of the basic field. To the group in charge of AVM, VM, combined type and syndrome, plastic surgeons and radiologists were mainly assigned. To the group in charge of CM and infantile hemangioma, plastic surgeons and dermatologists were mainly assigned. To the group in charge of the lymphatic system, pediatric surgeons, plastic surgeons and pediatricians were mainly assigned. The reviews of the guidelines were also prepared by those selected from each group. Pathologists and molecular biologists were in charge of the reviews of the basic fields.

### Preparation process

The guidelines were revised following the *Minds Handbook for Clinical Practice Guideline Development 2014* and *Minds Manual for Clinical Practice Guideline Development Ver. 1.0–2.0*.

Clinical questions were decided based on the following important clinical issues: (i) efficacy of resection; (ii) efficacy of sclerotherapy/embolization; (iii) efficacy of drug therapy, laser therapy, radiotherapy and other conservative treatment; (iv) differences in appropriate treatment due to the location of lesions; (v) differences in appropriate treatment among symptoms; (vi) appropriate timing of treatment and tests; and (vii) pathological diagnosis deciding the diagnosis.

For document retrieval, key words for published work searches were set for each CQ and work published from 1980 to the end of September 2014 was searched in PubMed, Cochrane Library and Japana Centra Revuo Medicina (JCRM) databases. A published work search was requested from the Japan Medical Library Association. For decisions on CQ and recommendations lacking evidence or having weak evidence, discussion and agreement in the preparation group were reflected.

The strengths of evidence and recommendations acquired by SR were determined following the Minds technique as described below and this follows the GRADE guidelines preparation method.^7^, ^8^

Determination of the strength of evidence of the body of evidence (Table 1)

**Table 1:** Recommendation grade and definition of the strength of body of evidence in evaluation of systematic review

Recommendation grade
1: strongly recommended
2: weakly recommended (suggested)
Definition of the Strength of Body of Evidence in Evaluation of Systematic Review
A (strong): strongly confident of the estimate of effect
B (moderate): moderately confident of the estimate of effect
C (weak): limited confidence of the estimate of effect
D (very weak): very little confident of the estimate of effect

The strength of evidence of the body of evidence was determined according to the *Minds Handbook for Clinical Practice Guideline Development 2014*.

In the case of RCTs [randomized controlled trials], the score “A (strong)” is given at the start of evaluation, and the final score might be downgraded to B, C, or D, according to the results of evaluation of five items, including risk of bias, inconsistency in results, indirectness of evidence, data imprecision and high possibility of publication bias. In the case of observational studies, the score “C (weak)” is given at the start of evaluation, and five items lowering the strength are evaluated similarly as for RCTs. In addition, three items, including large effect with no confounding factors, dose–response gradient, and possible confounding factors, are weaker than actual effects increasing the strength are evaluated as well…

### Presentation of the strength of recommendations

The strength of recommendation was also determined according to the *Minds Handbook for Clinical Practice Guideline Development 2014* (Table 1).

The strength of recommendations is usually presented in two ways: “1”: strongly recommended, and “2”: weakly recommended (suggested). If the strength of recommendations cannot be determined by any means, it is occasionally presented as “no definite recommendation can be made.” Recommendations will be entered as follows by indicating the strength of evidence (A, B, C, D) with the strength of recommendations “1”: strong or “2”: weak.

### Finalization

Preparation of the draft guidelines was completed in December 2016 and review was requested from the Japanese Society of Plastic and Reconstructive Surgery, Japanese Dermatological Association, Japan Radiology Society, Japanese Society of Interventional Radiology, Japanese Society of Pediatric Surgeons and Japanese Society of Pathology between December 2016 and January 2017, and corrections were made based on the results of the reviews. In addition, from December 2016 to January 2017, the guidelines were disclosed on the home page of the Research Committee for “Intractable Vascular Anomalies” and public comments were collected. The draft guidelines were presented to two related patient organizations, the Patients Association of Vascular Anomalies and the Patients Association of Combined Vascular Malformations, and comments were received. Based on these, the draft guidelines were revised and CQ, recommendations and explanations were completed. It was finalized in March 2017.

## RESULTS

### CQ and recommendations

#### CQ1: What are the guidelines for the time to begin treatment for AVM?

*Recommendation*: It is necessary to judge the time to begin endovascular or surgical treatment for AVM individually by evaluating the stage of symptoms and lesion extent and in consideration of the risk of complications.

*Strength of recommendation*: 2 (weak).

*Evidence*: D (very weak).

*Comments*: As a result of primary screening, 92, three and 27 papers were extracted from PubMed, JCRM and Cochrane Library, respectively, and, as a result of secondary screening, 37 and three papers were extracted from PubMed and JCRM, respectively. However, as all of these references were observational or case series studies, the strength of evidence is rated as D (very weak).

There has been no report in which time to begin treatment for AVM in itself was the end‐point, and only some reports described the view on the time to begin treatment in the discussion. Therefore, as it is difficult to objectively evaluate the validity of the time to begin treatment, we determined whether guidelines can be derived from the patient age, lesion site, symptoms, clinical stage, effectiveness of treatment and frequency of complications in each report.

In the two reports on the treatment for AVM,^9^, ^10^ symptomatic AVM is primarily addressed, and treatment can be suspended (follow up) while the lesion remains asymptomatic. However, as AVM often progresses when left untreated, it is considered important to begin the treatment at an appropriate time depending on the stage of symptoms. In addition, as there is the tendency that the response rate decreases, and the complication rate increases, with progression of symptoms, some reports from particular pediatric institutions where patients are concentrated recommend early therapeutic intervention in relatively “early” or “mild” stages without waiting for progression of the disease.

Localized lesions may be radically treated by early intervention.^11^ Among endovascular procedures, the response (cure) rate tends to be high by ethanol embolization, but the complication rate is also high.^12^, ^13^ By surgery, localized lesions are unlikely to recur if they are completely resected, although adverse events, such as postoperative cicatrization/deformation or functional impairment, have been seldom discussed.^10^ On the other hand, in diffuse lesions, limitations of effectiveness, such as recurrence and persistence and the risk of treatment, are higher by both endovascular treatment and surgery, with the risks outweighing the benefits.^13^ It should also be considered that children, in particular, are not mentally ready to accept such invasive treatments.^14^

As discussed above, it is difficult to give guidelines for the time to begin treatment for AVM at present, and individual judgment is necessary depending on the symptom stage and lesion extent in consideration of the complication risk.

#### CQ2: Is recurrence (regrowth) after resection of AVM more frequent by wound closure with a skin graft than by reconstruction using a flap?

*Recommendation*: Whether recurrence (regrowth) is more frequent by wound closure with a skin graft compared with reconstruction using a flap is unclear.

*Strength of recommendation*: 2 (weak).

*Evidence*: D (very weak).

*Comments*: The number of references retrieved by searches using key words was 40 from JCRM, 75 from PubMed and zero from Cochrane, and 39 were extracted by secondary screening. For AVM of a certain size, reconstruction is necessary after resection, and wound closure by skin grafting or reconstruction using a flap is performed according to common reconstruction methods for tissue defects. The reports discussing reconstruction after resection of AVM that we could retrieve were all descriptive studies (case reports or case series studies). Therefore, the evidence level of all of these references is D (very weak).

The concept of a regulating flap,^15^, ^16^ that reconstruction using a free flap controls the recurrence or regrowth after resection of AVM, has been proposed. However, no report has evaluated whether free flaps^16^, ^17^, ^18^, ^19^, ^20^, ^21^, ^22^, ^23^, ^24^, ^25^, ^26^, ^27^, ^28^, ^29^, ^30^, ^31^, ^32^, ^33^, ^34^, ^35^, ^36^, ^37^ and other flap types^17^, ^19^, ^25^, ^28^, ^38^, ^39^, ^40^, ^41^, ^42^, ^43^, ^44^, ^45^, ^46^, ^47^, ^48^, ^49^ clearly prevent the recurrence or regrowth compared with skin grafts.^21^, ^23^, ^50^, ^51^, ^52^

According to the present knowledge on the recurrence or regrowth after resection of AVM,^15^, ^16^, ^39^, ^53^ whether AVM can be completely resected is important, and, concerning cases in which complete resection is difficult, it has been reported that the hemodynamics in the residual lesions contribute to the recurrence and regrowth, and that they can be controlled by a flap with rich blood flow.

#### CQ3: Is proximal ligation/coil embolization of the feeding artery of AVM effective?

*Recommendation*: The therapeutic effect of ligation/coil embolization of the feeding artery on the proximal side may be poor, and the possibility of recurrence may be high. In addition, in the event of recurrence, treatment may become difficult due to the development of collateral vessels. Therefore, these procedures are recommended to be avoided, in principle.

*Strength of recommendation*: 2 (weak).

*Evidence*: D (very weak).

*Comments*: As a result of secondary screening, one and 14 papers were extracted from PubMed and JCRM, respectively, and were reviewed. As a result, all of these papers were case reports. In addition, six papers retrieved by manual searches were also reviewed, but they were all case series studies at the maximum. Therefore, the strength of evidence as a collection of published work concerning this CQ is D (very weak).

To summarize the evaluation of this collection of papers, AVM was treated by proximal ligation/coil embolization of the feeding artery, but as there have been reports of recurrence following the development of collateral channels (reports of cases of the occurrence of unfavorable situations), the treatment is recommended to be avoided, but the evidence level of this recommendation is low as mentioned above.

The objective of embolization for AVM is obliteration of the nidus, and embolization at or near the nidus is necessary as much as possible. If ligation/coil embolization of the feeding artery is performed on the proximal/central side, the nidus is not obliterated, and the development of several collateral channels is promoted. In many cases, the collateral channels are thin, complicated and markedly tortuous, and transcatheter treatment is often difficult.

Wu *et al.* 14 performed proximal ligation in nine of 29 patients treated for AVM of the auricle but the condition was exacerbated in all patients with eight requiring auricular resection and one requiring additional treatment. They excluded proximal ligation from treatment options for AVM, because it makes subsequent transcatheter treatments difficult.

Slaba *et al.* 54 evaluated 25 patients with AVM of the tongue and reported that three of the 12 symptomatic patients who underwent ipsilateral external carotid artery ligation at another facility showed marked development of collateral channels.

Other reports include those of a case in which a large number of collateral channels developed as a result of ligation of the feeding artery for AVM of the shoulder with serious complications including high‐output heart failure;^55^ three cases in which proximal ligation/embolization was performed for AVM of the limbs and pelvis, collateral vessels developed, but the condition was controlled by multidisciplinary treatment consisting of transcatheter treatment and direct puncture sclerotherapy;^56^ and several cases in which external carotid artery ligation was performed for AVM of the head and neck, but the subsequent treatment was difficult.

Suyama *et al.* 35 reported a case in which AVM of the auricle, treated by proximal embolization using coils and gelatin sponge, recurred and was treated again by ligation at a proximal part of the artery, but the lesion recurred again. Also, Aikawa *et al.* 57 reported a case of intrapelvic AVM that underwent coil embolization of the left ovarian artery and left internal iliac artery but showed little change in the area of the nidus or the state of arterial or venous dilatation. In addition, Yamamoto *et al.* 58 reported a case in which transarterial embolization (TAE) was performed for AVM of the mandible through the maxillary artery, facial artery, lingual artery and ocular artery, but was not effective due to the development of collateral channels from the internal carotid artery and vertebral artery.

As observed above, it is recommended not to select proximal/central ligation/coil embolization as a treatment for AVM. However, AV fistulas with direct connection of a large artery and a vessel may be treated by coil embolization if the shunt area is directly accessible with a catheter. Proximal coil embolization may also be accepted as preoperative embolization, but careful evaluation of its indications is necessary, and embolization at a site near the shunt is necessary to leave room for catheter insertion in the event of future recurrence.

#### CQ4: What is the appropriate timing for embolization before resection of AVM?

*Recommendation*: It is recommended to perform resection within 3 days (72 h) after embolization. If the interval is prolonged, the risk of massive intraoperative hemorrhage may increase due to recanalization of the embolized vessel and development of collateral channels. In addition, surgery has been reported to be made difficult by enlargement of the lesion after embolization.

*Strength of recommendation*: 2 (weak).

*Evidence*: D (very weak).

*Comments*: While it is difficult to generalize the therapeutic approach as it varies with the affected area and extent of the lesion, there were a few reports that preoperative embolization was useful for the treatment of AVM of the head and neck region.

As a result of secondary screening, 10 and three papers from PubMed and JCRM, respectively, were reviewed. All of the papers selected by this screening procedure were case reports or case series, and the strength of evidence is D (very weak). Mentions about the timing of preoperative embolization and volume of hemorrhage also varied among the papers. Although it is difficult to draw a conclusion, among the papers that mentioned specific timing of preoperative embolization and volume of hemorrhage, Deng *et al.* 59 performed embolization within 48–72 h before surgery in 16 patients with maxillofacial AVM and reported that the volume of hemorrhage was 200 mL or less in all patients and that there were no complications. Erdmann *et al.* 60 performed embolization within 24 h before surgery in four patients with head and neck AVM, and the lesion was resected with hemorrhage of 100 mL or less in three. It is recommended to perform resection within 72 h to prevent increases in difficulty of resection due to inflammation after embolization.

There have also been reports that embolization was performed intraoperatively or within a few days before surgery, resulting in decreases in the volume of hemorrhage or favorable long‐term outcomes. Most papers reported no or only mild complications, but as for relatively severe complications, Goldberg *et al.* 61 reported temporary visual impairment in two of the three patients with orbital AVM.

Factors that affect the appropriate timing of preoperative embolization include recanalization of the target vessel, development of collateral channels, and swelling and reactive changes after embolization, which make surgery difficult. To avoid the effects of these phenomena, many papers supported relatively early resection, namely within 72 h after embolization. Clinically, also, there is no benefit in taking a long interval, and it is considered valid to recommend resection within 72 h of embolization.

In conclusion, adequate control of hemorrhage may be achieved with fewer complications by performing vascular embolization within a few days before surgery, but no sufficient evidence that supports this view has been provided.

#### CQ5: What treatments are appropriate for maxillomandibular AVM?

*Recommendation*: Although surgery alone is not recommended, a combination of surgery with endovascular embolization (including sclerotherapy) can be recommended depending on the case.

Radiotherapy is not recommended.

Endovascular embolization (including sclerotherapy) alone or as a preoperative treatment can be recommended.

*Strength of recommendation*: 2 (weak).

*Evidence*: D (very weak).

*Comments*: AVM of the maxilla and mandible is a rare disorder. Most of the published works are reports of a small number of cases except a few case series reported from some special institutions. Only five reports^62^, ^63^, ^64^, ^65^, ^66^ of a series of 10 or more cases were retrieved by the search of PubMed. Because there is no cohort study or randomized trial comparing various treatments, strong evidence regarding the best treatment is lacking.

Maxillomandibular AVM may involve the maxilla, mandible or both, and it often presents with massive oral hemorrhage around the age of 10 years when milk teeth are lost, but may also be detected due to swelling of the soft tissue.

According to Persky *et al.*,^62^ embolization alone resulted in cure in 42%, improvement in 16% and stabilization of symptoms in 23% of the 26 patients with a maxillomandibular AVM. Liu *et al.* 63 treated 25 patients by transarterial or transvenous embolization alone or in combination with curettage and reported anatomical cure in 14 and clinical cure in 21. Chen *et al.* 65 treated 15 patients by bone wax packing (BWP) alone in four, TAE + BWP in three, TAE + resection in four and TAE + radiotherapy + resection in four, and reported clinical cure in 14.

The following are considered as treatment options for AVM of the maxilla and mandible:

1. A: Surgical treatment
  - A‐1: Resection and reconstruction
  - A‐2: Curettage
  - A‐3: BWP
2. B: Endovascular embolization (including sclerotherapy)
  - B‐1: TAE
  - B‐2: Transvenous embolization
  - B‐3: Embolization by direct puncture
3. C: Combination of A and B
4. D: Radiotherapy

The published work is mostly about B, namely endovascular embolization (including sclerotherapy) alone, or surgical treatment after B. There was no report of case series of surgical treatment alone, but there was only one report^65^ of case series of surgery + radiotherapy. Surgery alone and radiotherapy are generally not recommended. Endovascular embolization is performed by various approaches including transarterial and transvenous routes and direct puncture, sometimes in combination. Concerning embolic agents, polyvinyl alcohol and Gelfoam^®^ (Pfizer, New York, NY, USA) are used for embolization as an adjuvant therapy immediately before surgery as they allow recanalization. Cyanoacrylate liquid embolic agents are considered effective for embolization performed preoperatively or alone in expectation of a long‐term occlusive effect.^64^, ^67^, ^68^ Coils are often used for transvenous embolization. Recently, there have been reports^69^, ^70^ that favorable outcomes were obtained by TAE using Onyx^®^ (Medtronic, Irvine, CA, USA), a non‐adhesive liquid embolic agent. Concerning sclerotherapy, there is a case series study of ethanol sclerotherapy alone, reporting relatively favorable outcomes.^71^ Infection and bone necrosis are frequent complications of embolization, and they may occur when an embolic agent, a foreign body, is injected into lesions that were in contact with the external environment due to direct puncture or hemorrhage. Surgical treatments as listed above are performed primarily after endovascular embolization. Invasive radical resection and reconstruction should be avoided at least as the initial treatment, because many lesions can now be controlled by endovascular embolization alone with the advancement of endovascular technique.

As mentioned above, endovascular embolization is performed using various approaches and embolic agents selected depending on the facility and patient. There are also a wide variety of surgical treatment options. Because the treatment may be performed by combining these options, AVM of the maxilla and mandible should be treated at the institutions where multidisciplinary treatment can be performed by experienced physicians.

#### CQ6: What treatments are appropriate for AVM of the fingers?

*Recommendation*: Although embolization or sclerotherapy are effective because they alleviate symptoms, such as pain, sufficient evaluation is necessary because of the risk of finger necrosis and nerve damage. In surgical resection, total resection is recommended, because partial resection is likely to result in enlargement of the lesion. Occasionally, the disorder results in finger amputation.

*Strength of recommendation*: 2 (weak).

*Evidence*: D (very weak).

*Comments*: As a result of primary screening, 38, 16 and 35 papers were retrieved from PubMed, Cochrane Library and JCRM, respectively. However, during secondary screening, many reports concerned AV shunts in dialysis patients and AVM at sites other than the fingers. Eventually, only 10 papers comprising three case series and seven case reports remained as references, and the evidence level is extremely low (D: very weak).

Arteriovenous malformation of the fingers is often difficult to treat, and treatments are likely to be ineffective, particularly when the lesion extends from the fingers to the palm. In addition, when AVM is localized in the fingers, complications are likely to occur after treatment.^72^ It is recommended to conduct treatment by a team from several departments including plastic surgery, vascular surgery and radiology.^73^ 3‐D computed tomography (CT) angiography is useful for preoperative examination.^74^ Because complete cure is difficult to obtain by embolization therapy, it is recommended to be performed for alleviation of symptoms such as pain only in symptomatic areas.^75^ In addition, as there is the possibility of re‐enlargement after embolization, it is recommended to periodically follow up the condition and repeatedly perform embolization each time symptoms appear.^73^ Surgical resection is necessary for permanent cure, and total resection is recommended as there is the possibility of re‐enlargement after partial resection.^76^, ^77^, ^78^ Reconstruction is occasionally necessary, but treatment may end in finger amputation. In this event, preoperative embolization or sclerotherapy is effective.^79^ The present review has fallen short of clarifying situations in which preoperative embolization is useful in fingers to which a tourniquet can be applied.

#### CQ7: What treatments are effective for painful VM?

*Recommendation*: Sclerotherapy and surgical resection, as well as conservative treatments, such as compression, oral aspirin and low‐molecular‐weight heparin, are reported to be effective depending on the site and size of the lesion and symptoms. Endovascular laser treatment, percutaneous cryotherapy and photodynamic therapy have also been suggested to be effective.

*Strength of recommendation*: 2 (weak).

*Evidence*: D (very weak).

*Comments*: As a result of the published work search, 54 reports in English and four in Japanese were retrieved by primary screening. Of these reports, 39 in English and four in Japanese were extracted by secondary screening. Many options were reported as treatments for pain associated with VM, but all of these documents were case series or case reports without comparison of treatments. Therefore, the evidence level was rated as very weak and the recommendation level as weak.

Pain is one of the major symptoms of VM. It may respond to conservative treatments that are relatively easy to manage, such as compression and oral aspirin, depending on the site and size of the lesion and symptoms. Particularly when pain is localized, surgery should also be considered. Relatively novel treatments, such as endovascular laser therapy, percutaneous cryotherapy and photodynamic therapy, have been reported to be effective for controlling local VM, and they have also been reported to be effective for the control of pain. Limb lesions accompanied by localized intravascular coagulopathy may be indications for low‐molecular‐weight heparin. Reports on various treatments are mentioned below.

1. Compression

Although there has been no report of comparative evaluation, compression is reportedly effective according to reviews by specialized medical facilities.^80^, ^81^, ^82^

- Oral aspirin

The published work is also limited, but the treatment has been mentioned in reviews.^80^, ^81^, ^82^ Nguyen *et al.* 83 reported that pain was alleviated in 17 (77%) of 22 patients in whom oral aspirin therapy was initiated for pain.

- Sclerotherapy

Sclerotherapy has often been performed using ethanol or polidocanol. The published work concerning other sclerosing agents is scarce, and their effectiveness remains largely unclear.

Each sclerosing agent is commented on below.

(i) Ethanol

Shireman *et al.* 84 reported remission in six of 12 patients, and Rimon *et al.* 85 reported alleviation or remission in 14 patients with painful VM (including eight with lower limb lesions) except in four of those with lower limb lesions. Marrocco‐Trischitta *et al.* 86 reported that pain was resolved in both (100%) of two women with external genital lesions.

Concerning the use of ethanol, Suh *et al.* 87 reported alleviation to 50% or less of the pretreatment state according to a VAS in 12 (71%) of 17 patients who underwent sclerotherapy using its mixture with lipiodol, and Dompmartin *et al.* 88 reported 37 patients who underwent sclerotherapy using its mixture with ethylcellulose. According to Schumacher *et al.*,^89^ also, 77 patients underwent sclerotherapy using ethylcellulose ethanol in a multicenter study, and significant improvement compared with the pretreatment state was observed in all patients.

(ii) Polidocanol (including foam sclerotherapy)

Mimura *et al.* 90 reported remission in six, alleviation in four and no change in one of 11 patients with painful VM, and remission in 12 (41%), alleviation in 14 (48%), no change in two (7%) and exacerbation in one (3%) of 29 patients in another study.^91^ Cabrera *et al.* 92 treated 50 patients (including 15 with Klippel–Trenaunay syndrome) using a foamed sclerosing agent and reported remission in 25 (50%) and alleviation in 14 (28%). Marrocco‐Trischitta *et al.* 86 reported resolution of pain in all three women with external genital lesions.

(iii) Ethanolamine oleate

Ozaki *et al.* 93 reported remission in two (20%) and alleviation in eight (80%) of 10 patients.

(iv) Sodium tetradecyl sulfate (STS)

Krokidis *et al.* 94 reported alleviation of pain in four of five women with external genital lesions.

- Surgical resection

Enjolras *et al.* 95 performed surgical resection in seven of 13 patients with VM involving a wide area including the knee joint and reported alleviation of pain in five (71%). Steiner *et al.* 96 reported alleviation to 50% or less of the pretreatment state by a VAS in 24 (89%) of 27 patients with background pain and 12 (92%) of 13 patients with acute episodic pain. In addition, Noel *et al.* 97 performed surgical resection and compression therapy for VM of the lower extremities in 20 patients with Klippel–Trenaunay syndrome and reported disappearance of pain in 18 (90%) (mean follow‐up period, 63 months).

- Endovascular laser therapy

Sidhu *et al.* 98 and Lu *et al.* 99 reported alleviation of pain in all eight and 51 lesions in six and 33 patients, respectively. Liu *et al.* 100 also reported marked responses in 46 (35%), responses in 84 (63%) and no change in three (2%) of 133 patients.

- Low‐molecular‐weight heparin

According to Mazoyer *et al.*,^101^ only low‐molecular‐weight heparin was effective when VM were complicated by localized intravascular coagulation, resulting in disappearance of pain.

- Percutaneous cryotherapy

Cornelis *et al.* 102, 103 reported remission of pain in a report of one case (observation period, 2 months) and a report of four cases (observation period, 6 months).

- Photodynamic therapy

Betz *et al.* 104 reported remission in two and alleviation in one of three patients.

#### CQ8: Is laser therapy effective for VM?

*Recommendation*: With appropriate selection of the type of laser according to the site, size and symptoms of the lesion, laser therapy can be effective for the treatment of VM. It is recommended to evaluate whether the net benefit by laser therapy is worth the cost and resources by comparison with other treatments such as sclerotherapy and surgical resection.

*Strength of recommendation*: 2 (weak).

*Evidence*: C (weak).

*Comments*: VM is a lesion that has been called cavernous hemangioma, and it causes pain, functional impairment and cosmetic defect depending on the affected site. In addition to conventional resection of the lesion, sclerotherapy has been widely performed in recent years. While reports of laser therapy for VM have increased, there have not been prospective studies (PS) comparing the results of laser therapy with those of surgery or sclerotherapy, among types of laser equipment different in wavelength, or using the same equipment type but changing the irradiation method or parameter setting. We analyzed 134 papers extracted by primary screening and 98 papers extracted by secondary screening. Concerning more than 30 cases, the answer to the CQ was based primarily on seven reports summarizing the methods and sites of treatment and benefits and harms derived from treatment (decrease in size of the lesion, alleviation of symptoms, complications).

In the facial skin, pigmentation and scar formation after irradiation can be serious treatment‐related complications compared with unexposed areas. In the airway and digestive tract, the mass effect of the lesion and chronic bleeding from the lesion can be causes of serious symptoms. Thus, as the goal to achieve varies with the anatomical site of the lesion, we reviewed the published work by the anatomical site (the neurosurgery field was excluded). For this reason, we also extracted benefits and harms of treatment from the text of the reports of less than 30 cases. The relevant departments surveyed included ear, nose and throat, dental and oral surgery, gastrointestinal surgery, ophthalmology, plastic surgery and dermatology, and secondary screening overviewed laser therapy for VM and vasodilatory lesions.

**Figure 1:**
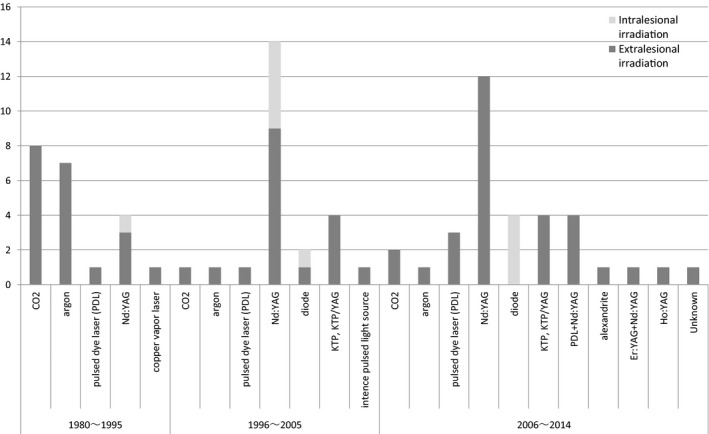
Reports of laser therapy for hemangiomas/vascular malformations (primarily venous) in various periods and types of laser used in the reports (CQ8). CO_2_: 10 of 11 reports recommend the use for surgical resection. Argon: there are four reports of mixed treatments for CM and VM. Neodymium:yttrium–aluminum–garnet (Nd:YAG): there are 12 reports of its use in combination therapy with surgery, sclerotherapy or other lasers. Alexandrite: there is only one report summarizing cases treated with alexandrite in combination therapy with other lasers. CQ, clinical question; Er:YAG, erbium:yttrium–aluminum–garnet; Ho:YAG, holmium:yttrium–aluminum–garnet; KTP, potassium titanyl phosphate laser; YAG, yttrium–aluminum–garnet.

When a new laser instrument is developed and put into use, reports of therapeutic results using the equipment are presented. The types of laser used for treatment varied widely. The types of laser that have been reported are summarized chronologically in a graph (Fig. 1). While the type of laser with more reports is not necessarily more effective, the graph is considered to reflect tendencies of laser types that are established and gain favorable appraisal or no longer in use.

Because dye laser used for the treatment of port‐wine stain (wavelength, 595 nm) uses hemoglobin as the observer/heater, photothermal conversion occurs efficiently in the blood vessel, and the thermal energy reaches endothelial cells.^105^ However, its optical penetration depth is shallow, being approximately 1 mm in both the skin and mucosa.^105^ However, in an erbium:yttrium–aluminum–garnet (Er:YAG) laser with a longer wavelength (1064 nm), the optical penetration depth is approximately 3 mm in the skin and approximately 6 mm in the mucosa.^105^ Although an neodymium:yttrium–aluminum–garnet (Nd:YAG) laser is advantageous compared with dye laser for the treatment of deep lesions, heat is generated also in perivascular tissues, because light is converted to heat as it is absorbed by water contained in the skin and mucosa.

The target of laser treatment for VM is the endothelium of morbidly dilated blood vessels. There is no light that is specifically absorbed by endothelial cells and emits heat. Satisfactory therapeutic results cannot be expected unless treatment is performed by selecting the laser type and modifying the irradiation method based on the understanding of such principles and limitations of phototherapy.

Concerning small VM of the mucosa, tongue, lips and glans penis, in which scar formation after treatment poses no serious problem, there are a number of reports^106^, ^107^, ^108^ that lesions were resolved by treatment using Nd:YAG laser. There have also been cases in which favorable results were obtained by treatment of anemia due to gastrointestinal bleeding^109^ and of symptoms, such as airway obstruction, due to the mass effect of the lesion.^110^ While transient purpura and swelling after treatment are unavoidable, they often resolve rapidly.^99^ Modifications of the irradiation setting and method are necessary to obtain satisfactory results and avoid serious complications, such as peroneal neuropathy^100^ and pigmentation and scar formation of the facial skin,^106^, ^108^ and we must learn from the experience of experts.

Neodymium:yttrium–aluminum–garnet laser irradiation by insertion into the lesion under ultrasound guidance has begun to be performed as a treatment to avoid damaging important organs and nerves,^98^, ^99^, ^100^ therapeutic experience using this technique has been accumulated, and detailed records and reports have been presented. At present, the results have been satisfactory in terms of safety and efficacy, and standardization of the procedure is anticipated.

#### CQ9: Is sclerotherapy effective for VM?

*Recommendation*: Sclerotherapy for VM is effective for alleviating symptoms and reducing the size of the lesion and is recommended.

*Strength of recommendation*: 2 (weak).

*Evidence*: D (very weak).

*Comments*: VM is a lesion that used to be called cavernous hemangioma or intramuscular hemangioma and differs from infantile hemangioma. VM poses problems, such as pain, swelling and functional impairment, and has been treated conventionally by surgical resection. In Western countries, percutaneous sclerotherapy has a long history. In 1989, Yakes *et al.* 111 reported ethanol sclerotherapy for VM, and the treatment has since been performed worldwide. Recently, sclerotherapy, which is mildly invasive, permits functional and morphological preservation, and can be performed repeatedly, has become widely used. However, as of 2016, sclerotherapy is not covered by medical insurance in Japan. In addition, there has been no RCT on the usefulness of sclerotherapy for VM compared with surgery or placebo.

As a result of secondary screening, 76, three and three papers were extracted from PubMed, Cochrane and JCRM, respectively. They include three semi‐RCT, but randomization and blinding were insufficient, and their quality as an RCT was low. Also, the theme evaluated by all these RCT was “comparison of sclerosing agents in sclerotherapy”, and none compared sclerotherapy with other treatments. Therefore, control groups related to this CQ were not established, and their contribution as a whole is weak. The other published work was all case reports or case series, and the evidence level is D (very weak). As mentioned above, while the evidence level is low, most of the studies reported alleviation of symptoms and regression of lesions in a high percentage (70–90%) of the patients, suggesting the usefulness of sclerotherapy.

The sclerosing agents used included absolute ethanol, polidocanol, ethanolamine oleate, STS and bleomycin. Polidocanol is approved as a sclerosing agent for lower limb varices and esophageal varices, and ethanolamine oleate as a sclerosing agent for esophageal varices. STS is not marketed in Japan. Each sclerosing agent has characteristic complications. Recently, injection of polidocanol, STS, as a foam by mixing with CO_2_ or air has been increasingly performed. Sclerotherapy using ethanol is often performed under general anesthesia, but sclerotherapy using polidocanol or ethanolamine oleate can be performed under local anesthesia.

Three RCT have been reported as studies that evaluated differences in therapeutic effect according to the sclerosing agent. However, randomization and blinding are insufficient, and their quality as an RCT is low. In addition, the theme evaluated in these RCT was “comparison of sclerosing agents in sclerotherapy” rather than comparison with other treatments.

Although the evidence level is low, there have been a few case series that reported the usefulness of sclerotherapy, and a wide variety of sclerosing agents including ethanol, polidocanol, ethanolamine oleate, STS and bleomycin were used. Among studies with a relatively large number of patients, there is a report^112^ that sclerotherapy using ethanol in 87 patients with craniofacial VM resulted in a 75% or more decrease in size in 23 (32%) and a 25–75% decrease in size in 37 (52%). The results of sclerotherapy using polidocanol in 50 patients with VM were excellent in 19, good in 16, moderate improvement in 13 and unchanged or worse in two.^92^ The results of sclerotherapy using ethanolamine oleate performed in 83 patients, who were mostly children, were complete remission of symptoms in 79 lesions and significant alleviation in six lesions.^113^ Sclerotherapy using STS resulted in subjective improvements in 174 (85.3%) of 204 patients.^114^ The results of sclerotherapy using bleomycin were complete cure in 185 of 260 patients, marked improvement in 44 and some improvement or no change in 31.^115^ In addition, regarding the size of the lesion, a very satisfactory decrease was achieved in 104, and a satisfactory decrease was achieved in 10 of 120 patients.^116^

Papers that evaluated the types of VM that are likely to respond to sclerotherapy include those by Goyal *et al.*,^117^ Yun *et al.*,^118^ Mimura *et al.*,^91^ Rautio *et al.*,^119^ Lee *et al.*,^112^ Yamaki *et al.* 120 and Nagao *et al.* 121 Types of lesions that were likely to be sclerosed were reported to be well‐defined small (≤5 cm) lesions by Goyal *et al.*;^117^ those in females, lesions showing no or delayed delineation of the draining vein and lesions well‐defined on magnetic resonance imaging (MRI) by Yun *et al.*;^118^ small lesions, well‐defined lesions and lesions that show prolonged drug retention by Mimura *et al.*;^91^ localized lesions by Lee *et al.* 112 and Yamaki *et al.*;^120^ and slow flow type lesions by Nagao *et al.* 121 Nomura *et al.* 122 evaluated the therapeutic effect according to the degrees of functional and gross improvements and reported that the therapeutic effect was greater in head and neck and trunk lesions than in the upper or lower limb lesions. Moreover, Rautio *et al.* 119 reported that the treatment‐related improvement in the quality of life was higher when the lesion did not involve the muscle or was 5 cm or less in size.

Various complications ranging from mild complications, such as transient neuropathy and local inflammation, to serious ones, such as myopathy, skin necrosis and deep venous thrombosis/pulmonary embolism, have been reported. In sclerotherapy using ethanol or polidocanol, particularly serious life‐threatening complications have been reported. Qiu *et al.* 123 reviewed the published work concerning sclerotherapy for VM and reported that shock and pulmonary embolism occurred in 0.19% each of 522 patients who underwent sclerotherapy using ethanol and that ethanol was used at 1 mL/kg in those who developed shock. They also reported that a decrease in blood pressure/bradycardia was noted in 0.61% of 163 patients who underwent sclerotherapy using polidocanol but that its differentiation from vagus nerve reflex was clinically difficult. Wong *et al.* 124 reported a case of shock after sclerotherapy using 0.86 g/kg ethanol but could be saved. Tachibana *et al.* 125 reported that two patients (1.1%) developed pulmonary embolism and that the amounts of ethanol used were 0.71 and 0.16 mL/kg. Concerning sclerotherapy using polidocanol, also, cardiac arrest in children has been reported by authors including Marrocco‐Trischitta *et al.* 126 and Shimo *et al.*,^127^ who used 4 mL of 1% polidocanol (bodyweight, 20 kg) and 10 mL of 3% polidocanol (15.6 kg), respectively.

In conclusion, sclerotherapy is generally considered effective for VM, but its problems are that the evidence level is low and that the procedure has not been standardized. In addition, serious complications that are rare but life‐threatening have been reported, and caution is needed in deciding the dose of the sclerosing agent.

#### CQ10: Are clotting abnormalities due to VM an indication for radiotherapy?

*Recommendation*: Radiotherapy should not be performed without careful evaluation because malignant neoplasm, growth disorders and functional impairment have been reported as late complications.

Many reports included both VM and vascular tumors in the subjects, which make it difficult to assess the therapeutic effects of radiotherapy.

*Strength of recommendation*: 2 (weak).

*Evidence*: D (very weak).

*Comments*: As a result of primary screening, six and two documents were retrieved from PubMed and JCRM. However, as a result of secondary screening, liver hemangioma was excluded, and 10 papers including the references from the previous guideline were reviewed. The reviewed papers were case series or case reports, and the evidence level of the published work as a whole is D (very weak).

While there have been reports that radiotherapy was performed for the treatment of vascular tumors and vascular malformations, it is difficult to judge whether the treatment was performed by determining the disorders.

According to many reports,^128^, ^129^, ^130^, ^131^, ^132^ radiotherapy has been performed to treat Kasabach–Merritt phenomenon. However, while there is no mention of Kasabach–Merritt phenomenon, there is a report^133^ of five cases in which giant hemangiomas accompanied by clotting disorders, thrombocytopenia, heart failure and bleeding were controlled by multidisciplinary treatment including radiotherapy.

Vascular tumors that cause Kasabach–Merritt phenomenon are considered to be kaposiform hemangioendothelioma or tufted angioma rather than infantile hemangioma.^134^ Because VM and infantile hemangiomas are included in other vascular tumors in the lesions described in these reports, they are not considered to be indications of radiotherapy for VM or infantile hemangiomas.

Schild *et al.* 128 reported 13 cases of symptomatic hemangioma (11 of which were pathologically diagnosed as cavernous hemangioma, but as the report is old, vascular tumors and vascular malformations were not distinguished and were probably included). Radiotherapy at 6.25–40 Gy was carried out in these 13 cases. The lesions were located in the limbs in five, face in two, vertebral bodies in three, pituitary fossa in one, sacrum in one and bladder in one. Note that organs that should be excluded in this CQ were included.

Of these patients, two (one each with a limb and facial lesion) exhibited Kasabach–Merritt phenomenon and showed normalization of clotting disorder (evaluated according to the platelet count and fibrinogen level) after treatment. However, they were aged 3 years and 5 months, and the lesions might not have been VM.

When the subjects were limited to patients with limb or facial lesions, complete response (CR) was observed in two, partial response (PR) in four and no response in one in terms of decrease in the lesion size, and CR was observed in four, PR in one and no response in two in terms of the control of symptoms.

A serious treatment‐related complication, which was unilateral visual impairment, was noted in one (14 Gy/8 fr).^128^ These problems have been recognized as late complications of radiotherapy for vascular tumors or vascular malformations; malignant neoplasms, such as breast cancer,^135^ thyroid cancer^136^ and vascular sarcoma,^137^ visual impairment mentioned above,^128^ and shortening of the lower limb and restriction of the joint motion range.^131^

According to Coldwell *et al.*,^137^ late complications of radiotherapy for hemangiomas in infancy include bruise and Stewart–Treves syndrome after the patients reach adulthood. Angiosarcoma is also observed. They reported that the median survival period was 24 months, and the 5‐year survival rate was approximately 10%, in those who developed angiosarcoma.

As observed above, the diagnosis was not confirmed in the reports that have suggested the effectiveness of radiotherapy, and its indications have not been specified. In addition, there have been a considerable number of reports of late complications due to radiotherapy. Thus radiotherapy should not be performed without careful evaluation.

#### CQ11: Is there difference in the effectiveness of dye laser treatment for CM according to the site of the body?

*Recommendation*: Dye laser treatment for CM is likely more effective in the face and neck region compared with other sites, and it is more likely to cause complications such as pigmentation in the limbs.

*Strength of recommendation*: 2 (weak).

*Evidence*: C (weak).

*Comments*: As a result of published work searches, 176 papers consisting of 139 from PubMed and 37 from JCRM were extracted. They included a few reports that were allegedly RCT but were not actual RCT. Therefore, a total of 26 papers consisting of 15 from PubMed and 11 from JCRM including case series with a large number (≥100) of relevant cases were selected by secondary screening. In addition, a total of 17 papers were adopted as references for the comments in the guidelines by adding three papers in English extracted by manual search to six from PubMed and eight from JCRM considered to be relevant or closely related to the CQ among those selected by secondary screening. Because there was no RCT, the evidence as a whole was rated as C (weak).

Concerning the effect of dye laser treatment for CM, most of the reports were about the effects for hemangioma simplex or port‐wine hemangioma in Japan and port‐wine stain abroad.

There have been a few papers^138^, ^139^, ^140^, ^141^, ^142^, ^143^, ^144^, ^145^, ^146^, ^147^, ^148^, ^149^, ^150^, ^151^, ^152^ that evaluated the therapeutic results of dye laser treatment according to the site in a small to relatively large number of patients. The laser equipment used varies from early dye laser to pulsed‐dye laser with adjustable pulse duration with a cooling system, and reports limited to variable‐pulse pulsed‐dye laser with a cooling system, which is widely used today, are extremely few.

According to many reports,^138^, ^139^, ^140^, ^141^, ^142^, ^143^, ^144^, ^145^, ^146^, ^147^, ^148^, ^149^ the response rate is higher in the face and neck region than in the trunk and limbs. In the face, it has been reported that the response rate is higher in the palpebral, forehead and temporal, and lateral buccal regions but is significantly lower in the territory of the second division of the trigeminal nerve (dermatome V2), and that the number of irradiations tends to increase in the midline region, frequently resulting in persistence of redness.^150^ There is a report^151^ that the response rate did not differ significantly among regions in the lower limb. While the number of patients was small, it has been reported that treatment of the foot involves stronger pain but was less effective than in the face but that the degree of patient satisfaction was relatively high.^152^

The incidence of complications of dye laser (bleb formation, depigmentation, pigmentation, scar formation) is reported to be low, being 1.7% in adults, 0.6% in children and approximately 1.4% in all patients even when all sites of the body are included, and no significant difference has been reported in the age at the beginning of treatment, Fitzpatrick skin type,^153^ site, number of treatments or irradiation energy between those who developed complications and those who did not, but complications may occur more frequently in the lower limbs.^154^ Moreover, there is a report^152^ that complications, such as pigmentation, depigmentation and atrophic scar, were observed more frequently in the lower limbs.

#### CQ12: Do CM recur after dye laser treatment?

*Recommendation*: Although the effectiveness of dye laser treatment for CM is established, the recurrence rate may increase with time after treatment.

*Strength of recommendation*: 2 (weak).

*Evidence*: C (weak).

*Comments*: As a result of published work searches, a total of 211 papers consisting of 149 from PubMed, 53 from Cochrane and nine from JCRM were retrieved. They did not include RCT, and a total of 30 papers consisting of 23 from PubMed and seven from Cochrane, which were mostly case reports and case series studies, were extracted by secondary screening. In addition, a total of 10 papers that were relevant and closely related to the CQ (including eight case series) consisting of seven from PubMed, two from Cochrane and one in English retrieved by manual search were adopted as references for the guidelines. Because there was no RCT, the strength of evidence of the group of published work concerning this CQ is C (weak).

Concerning papers that referred to “whether CM recur after dye laser treatment”, there are four retrospective studies^155^, ^156^, ^157^, ^158^ after treatment by pulsed‐dye laser (wavelength, 585 nm) with a cooling system, and the recurrence rate was 15.9–35%. Also, there is a report^155^ that the recurrence rate increased with time after treatment and was 3.1% after 1 year, 20.8% after 2 years, 40% after 3 years and 50% after 4 years. Therefore, it is necessary to treat CM with the recurrence after dye laser treatment in mind.

It is difficult to strictly distinguish whether the recurrence is generation of new dilated vessels after laser therapy or regeneration of blood vessels damaged due to treatment or re‐proliferation of remaining vessels. However, there have been reports that, in an experiment using mice, angiogenesis occurred in the process of wound healing at the site of irradiation in early recurrence^159^ and that, in an experiment using hamsters, complete treatment was difficult, and morbid vessels persisted, because coagulation was difficult to induce by dye laser irradiation in vessels ranging 2 μm or less to16 μm in diameter.^160^ While there is a report^161^ that genes affected by dye laser therapy early after treatment were identified, further evaluation is necessary to clarify their relationships with the recurrence.

Concerning the prevention of recurrence, there is a report^162^ that the recurrence‐free period was long in the patients treated with a variable‐pulse pulsed‐dye laser with a cooling system (wavelength, 595 nm), which is widely used today, and they were treated within 6 months after birth. In addition, there have been reports of animal experiments using rapamycin, which inhibits angiogenesis after laser irradiation,^159^, ^163^ and of prospective RCT using imiquimod,^164^, ^165^ and these treatments were considered effective for the prevention of recurrence. However, careful evaluation by large‐scale investigations, including the assessment of the safety concerning drugs, is considered necessary.

#### CQ13: Is dye laser irradiation for CM more effective as it is initiated at a younger age?

*Recommendation*: Laser therapy before the age of 1 year may be effective, and the earliest possible initiation of treatment is recommended as an option.

*Strength of recommendation*: 2 (weak).

*Evidence*: D (very weak).

*Comments*: Concerning the timing of treatment for CM, there is the opinion that early initiation of treatment is recommended because in young children the skin is thinner, so the depth of penetration is larger, the vascular wall is also immature, cure after laser irradiation is better, pigmentation is less and the irradiation area is small, so the treatment efficiency is higher. However, there is still controversy. As a result of secondary screening of past reports, six and one were extracted from PubMed and JCRM, respectively. While the papers selected by these screening procedures include two papers on PS as described below, their conclusions differed, and the evidence level is considered to decline when these references are reviewed together.

Oguri *et al.* 138 performed a non‐RCT by dividing children into those aged 0–12 months, 13–24 months and 25–36 months, and observed significant differences in the response rate combining “markedly effective” and “effective” among the groups. They also compared the response rate according to the age in months at the beginning of treatment in the 0‐year‐old group and reported that the response rate was higher as the treatment was initiated earlier. Furthermore, Nguyen *et al.* 166 divided their patients into those aged less than 1 year, those aged 1–6 years and those aged 6 or more years, and investigated the correlation between treatment response and age. They reported that those aged less than 1 year and lesions with a size of less than 20 cm^2^ located in the center of the face showed the best treatment response.

Among reports suggesting no difference in the therapeutic effect according to the age at the beginning of treatment, van der Horst *et al.* 167 studied 100 patients with untreated CM of the head and neck region prospectively and concluded from the results of colorimetry and clinical evaluation that there was no significant difference in the therapeutic effect of pulsed‐dye laser among the four groups in which the treatment was started at the age of 0–5, 6–11, 12–17 and 18–31 years. In the retrospective study of Katugampola *et al.*,^140^ also, comparison of four groups in which treatment was started at the age of 0–5, 6–12, 13–50 and 50 years or more showed no significant difference in the therapeutic effect.

Among the above reports, those that did not affirm the usefulness of early laser treatment were relatively old. Also, reports of Oguri *et al.* 138 and Nguyen *et al.* 166 indicated that laser therapy may be more effective in those aged less than 1 year. In addition, the effectiveness of laser clearly declines when the lesion is elevated or thickens with time. In consideration of “benefits” of early laser treatment and “harms”, which include the occasional necessity of general anesthesia for laser treatment around the eye in small children, the recommendation level was rated as 2D based on the consensus of this guidelines drafting committee.

#### CQ14: Is propranolol safe and effective for infantile hemangiomas?

*Recommendation*: If administrated under careful monitoring, oral propranolol therapy may be the first choice for the treatment of infantile hemangioma.

*Strength of recommendation*: 1 (strong).

*Evidence*: A (strong).

*Comments*:

1. Effectiveness

There was the serendipity that regression of hemangioma was induced in a child under steroid therapy with a giant infantile hemangioma by propranolol administrated for obstructive hypertrophic cardiomyopathy in 2008.^168^ Based on this report, oral propranolol therapy began to be used for the treatment of infantile hemangioma, and its high efficacy against alarming hemangioma/life‐threatening hemangioma in the proliferating phase and in patients with cosmetic problems, such as giant lesions in the face, those with ulcerated and hemorrhagic lesions and those who may develop functional impairment, has been demonstrated, resulting in its use (Hemangiol®, Pierre Fabre Dermatologie, Boulogne, France) as the first choice in Western countries. In addition, its effectiveness for the treatment of hemangiomas after the proliferating phase was also described. Moreover, a group of physicians used propranolol earlier due to cosmetic significance and at the request of the family even in cases of small or localized lesions, and it is also effective in such cases.

A total of 131 papers consisting of 25 from JCRM, 106 from PubMed and zero from Cochrane were extracted as related to the CQ, “Is propranolol safe and effective for infantile hemangioma?”, and they were subjected to primary and secondary screenings with reduction of hemangioma (effectiveness of propranolol) and treatment‐related complications (adverse effects) as outcomes. Twenty‐six papers,^169^, ^170^, ^171^, ^172^, ^173^, ^174^, ^175^, ^176^, ^177^, ^178^, ^179^, ^180^, ^181^, ^182^, ^183^, ^184^, ^185^, ^186^, ^187^, ^188^, ^189^, ^190^, ^191^, ^192^, ^193^, ^194^ most of which were RCT or observational studies, were adopted.

For example, Hogeling *et al.* 180 administrated placebo or propranolol at 2 mg/kg per day for 6 months with randomization to 40 patients aged 9 weeks to 5 years with infantile hemangiomas in the face or sites with the potential for disfigurement. They reported significant improvements in size, redness and elevation in the propranolol group. Elevated lesions disappeared in four of the 19 patients in the propranolol group but none of the 18 patients in the placebo group. As for adverse events, the trial was interrupted in one patient due to upper respiratory tract infection (URTI), and conditions including bronchiolitis, gastroenteritis, streptococcal infection, cool extremities, dental caries and sleep disturbance were observed.

Zaher *et al.* 181 observed 45 patients by randomly dividing them into 15 each treated by p.o. administration, topical application and intralesional injection of propranolol. Responses were observed in 60% in the oral group, 20% in the topical ointment group and 13.3% in the injection group. No major adverse events were noted, and the trial was discontinued in one in the oral group and three in the injection group due to inconvenience or pain of the treatment.

Malik *et al.* 182 randomly allotted 30 patients aged 1 week to 8 months to propranolol alone, prednisolone alone or both propranolol and prednisolone. They found that the mean initial response times were lower in the propranolol group than in the prednisolone group but that there was no clear difference between the propranolol + prednisolone group and propranolol alone group. All 10 patients in the propranolol group and nine patients in the corticosteroid group responded to the 3‐month treatment. However, adverse events were observed in two of the 10 patients in the propranolol group (asymptomatic hypoglycemia, insomnia) and nine of the 10 patients in the steroid group (cushingoid appearance, gastrointestinal upset), and were more frequent in the latter group.

Bauman *et al.* 183 performed a phase 2, investigator‐blinded, multicenter RCT in 44 patients aged 2 weeks to 6 months. Propranolol or prednisolone (2 mg/kg per day) was administrated p.o. until halted due to toxic effects or clinical response. During 4 months of treatment, no significant difference was observed between the two groups, for example, with regression of five of the six tumors in the corticosteroid group and nine of the 10 tumors in the propranolol group. For long‐term analyses, the effect of prednisolone appeared earlier. While the incidence of adverse events as a whole did not differ between the two groups, severe adverse events were observed in one of the 11 patients in the propranolol group but five of the seven patients in the prednisolone group, being significantly more frequently in the latter group.

Léauté‐Labrèze *et al.* 184 carried out an RCT in patients aged less than 4 months by comparing seven administrated and seven not administrated propranolol. Because color changes and softening were observed within 24 h, and the thickness and size of the lesions decreased within 4 weeks in the propranolol group, the treatment was considered useful for the prevention of scarring. No serious adverse effect was observed except asymptomatic mild decreases in heart rate and diastolic blood pressure.

There have also been comparisons between atenolol and propranolol and between laser and laser + topical propranolol.^185^, ^186^ In 2015, the largest RCT was published in the *New England Journal of Medicine*, also reporting that propranolol was significantly effective for hemangioma compared with placebo.^187^ Hemangioma showed complete or nearly complete resolution after 6 months of treatment in two (4%) of 55 patients in the placebo group and 61 (60%) of 101 patients in the 3 mg/kg per day propranolol group.

Furthermore, there have also been a few SR and meta‐analyses primarily of observational studies. Menezes *et al.* 188 reviewed 49 English‐language papers published between June 2008 and September 2010, and summarized six studies with 10 or more patients administrated propranolol (154 patients in total). Propranolol was administrated to infants with a mean age of 4.5 months at a dose of 2 mg/kg per day in 65% and 3 mg/kg per day in 25.3%. Two‐thirds of the patients were treated with propranolol alone. Recurrence was observed in 21% after treatment for a mean of 4.3 months, and adverse events including hypotension, somnolence, wheezing, insomnia, agitation, nightmare, cool hands, night sweat, gastroesophageal reflux disease and psoriasiform rash appeared in 18.1%.

Marqueling *et al.* 189 reviewed the therapeutic results in 1264 patients (including 806 girls) in 41 reports published from 2008 to 2012 retrieved from Medline and Cochrane. The treatment was initiated at a mean age of 6.6 months at 2.1 mg/kg per day and continued for a mean of 6.4 months. The overall response rate was 98%, and the treatment was also effective in clinically problematic areas such as the face (100%), airway (100%), periorbital (98%), head and neck region (97%), and parotid gland (82%). However, recurrence was observed in 17% after treatment. Adverse effects were noted in 371 of 1189 patients. Changes in sleep (136 patients) and acrocyanosis (*n* = 61) were the most frequent among them, and hypotension was observed in 44, bradycardia in nine and hypoglycemia in four as serious complications. In conclusion, the grade of recommendation was 1, quality of evidence is A and propranolol was recommended as the first‐line drug for complicated infantile hemangiomas. Regarding adverse effects, the grade of recommendation was 1, and the quality of evidence was A or B. While serious adverse effects may be observed, their frequency is low, and they can be usually avoided by proper monitoring at initiation of treatment.

Xu *et al.*,^190^ on the other hand, evaluated volume changes, improvement in overall appearance, visual function and adverse effects using 15 online databases. The data of 419 cases were analyzed, but meta‐analysis was not performed because of the wide differences among studies. Some studies showed superiority of propranolol compared with corticosteroid in reducing volume and improving the overall appearance. No marked difference was noted in adverse effects or visual function.

In addition, in a meta‐analysis of 16 studies (2629 cases) and 25 studies (795 cases) published in 1965–2012, 69% of the patients responded to 12‐month corticosteroid therapy, but the response rate to propranolol was 97% with a significant difference.^191^

In periorbital hemangiomas, the response rate to propranolol was found to be significantly higher than that to corticosteroid by meta‐analysis of papers published before 2013,^192^ and propranolol showed the strongest effect against airway hemangiomas compared with steroid, CO_2_ laser and vincristine on meta‐analysis.^193^, ^194^

From these observations, we concluded that propranolol was significantly more effective than placebo and to be similarly effective compared with corticosteroid. Concerning the safety, propranolol is considered to have significantly fewer adverse effects than corticosteroid. Because there have been several RCT and SR or meta‐analyses directly related to this CQ, the evidence level is considered to be extremely high.

- Meta‐analysis

Regarding the effectiveness and adverse effects of propranolol, a large number of SR and meta‐analyses based on observational studies are already present in the above 26 papers. We, therefore, used only four reports^180^, ^182^, ^183^, ^187^ on interventional studies for meta‐analysis.

As a result of meta‐analysis, regarding “tumor reduction”, it was found that propranolol had significantly stronger reducing effects than placebo and that it had a stronger reducing effect, which, however, was not significant, compared with corticosteroid. Concerning “complications”, propranolol was compared with steroid and was shown by two RCT to have significantly fewer adverse events than corticosteroid. Because this meta‐analysis found significantly stronger reducing effect of propranolol compared with placebo and in fewer complications compared with steroid, and because our results were similar to those of SR of many existing observational studies considered to have high‐quality evidence, we considered that there was a major tendency in this CQ and judged the evidence level as A.

- Estimated action mechanism

Beta‐blockers have a wide range of actions on the blood vessels and vascular endothelium, and have diverse actions on cell proliferation and vascular remodeling. Thus, the mechanism of action of propranolol on infantile hemangiomas is still unclear. In vascular endothelial cells, propranolol is considered to induce vascular contraction by suppressing NO production, inhibit renin production, control angiogenesis by regulating the expression of vascular endothelial growth factor·basic fibroblast growth factor·matrix metallopeptidase (MMP)2/MMP9, and induce apoptosis, but it may also affect pericytes and hemangioma stem cells.^195^, ^196^, ^197^

- Adverse events associated with propranolol in children

In conducting propranolol therapy, it is necessary to have knowledge of possible adverse effects, their symptoms and their management. In addition, as there are also preventive measures for, and points of attention about, adverse effects and the timing for discontinuation of propranolol, sufficient explanation to the patients and their families is essential.

Adverse events that have been reported include sleep disorders, peripheral cyanosis, hypotension (symptomatic, asymptomatic), bradycardia (symptomatic, asymptomatic), hypoglycemia, respiratory disorders, gastrointestinal disorders and mental disorders. Severe cases that require interruption of treatment are few, but particular caution is needed regarding the following points.^189^, ^196^, ^197^, ^198^, ^199^, ^200^

1. Because there is the risk of hypoglycemia, the patient should be fed before and after propranolol administration. If the patient cannot be fed, or is vomiting, for some reason, the administration should be suspended.
2. Because propranolol has cardiovascular adverse effects, such as hypotension and bradycardia, interviewing for the past history and familial history, examination and electrocardiography are recommended before treatment. Even if no abnormality is noted on these examinations, hypotension and bradycardia may occur during treatment. In such cases, interruption of the administration is necessary.
3. Propranolol is contraindicated for bronchial asthma as it causes bronchial contraction due to its β2‐blocking action. Caution is also necessary in patients who have been suspected to have bronchial asthma.

#### CQ15: What treatments are effective for ulcer formation in infantile hemangioma?

1. Propranolol

*Recommendation*: The administration of propranolol is recommended for ulcer formation.

*Strength of recommendation*: 2 (weak).

*Evidence*: C (weak).

- Topical administration of antibiotics

*Recommendation*: Topical and systemic administration of antibiotics is recommended for ulcer formation.

*Strength of recommendation*: 2 (weak).

*Evidence*: D (very weak).

- Dressings

*Recommendation*: The use of dressings is recommended for ulcer formation.

*Strength of recommendation*: 2 (weak).

*Evidence*: D (very weak).

- Laser therapy

*Recommendation*: Although laser therapy may be effective in some patients with ulcer formation, the evidence is not considered sufficient at present.

*Strength of recommendation*: 2 (weak).

*Evidence*: D (very weak).

- Systemic administration of steroid

*Recommendation*: Systemic administration of steroid is recommended not to be performed for ulcer formation.

*Strength of recommendation*: 2 (weak).

*Evidence*: D (very weak).

- Platelet‐derived growth factor preparations

*Recommendation*: The accumulation of cases is insufficient for the judgment of the recommendability of the use of platelet‐derived growth factor preparations for ulcer formation.

*Strength of recommendation*: No recommendation.

*Evidence*: D (very weak).

*Comments*: Concerning this CQ, 42 papers in Japanese and 156 in English were retrieved. As a result of their primary screening, 47 papers were submitted to secondary screening for this CQ. None of them were about studies with a high level of evidence, such as RCT, and they were all retrospective studies, case series or case reports.

As a result, 15 papers in English were adopted, and the evidence level was C for propranolol alone, because of the presence of a prospective controlled trial, but D for other treatments, because the related papers were case reports or case series.

According to cross‐sectional analysis in a multicenter prospective cohort study in 1096 cases of infantile hemangioma by Chamlin *et al.*,^201^ it was complicated by ulcer, which was or was not bleeding, in 173 (15.8%), the median age of the patients was 4.0 months (standard deviation, 8.5; mean, 6.6 months), and the age at the first examination was significantly lower in patients with ulcerated hemangioma (median, 3.5 months; mean, 3.98 months) than in those with non‐ulcerated hemangioma.

By the site, ulcer formation was observed in 21 (30%) of 71 patients in the lower lip, 25 (25%) of 100 patients in the neck and 46 (50%) of 93 patients in the perianal/perigenital area, and the frequency was statistically lowest in the upper eyelid (*P* = 0.0140).

Ulcer formation was observed more frequently in mixed or segmental hemangiomas. Bleeding was noted in 78 lesions (41%) and was mild in 56 (29%), moderate in 11 (6%) and severe in four (2%). Severe bleeding occurred in three lesions in the limbs and one lesion in the face, and bleeding occurred in two cases at home. Two cases required blood transfusion by hospitalization, because they showed symptoms due to serious bleeding. Of the ulcerated hemangiomas, 67 (35%) were in the proliferating phase.

Ulcerated hemangiomas required treatment (odds ratio [OR], 6.86; 95% CI, 3.70–12.71; *P* < 0.0001), and non‐ulcerated hemangiomas were observed (OR, 19.01; 95% CI, 11.23–28.88; *P* < 0.0001). Ulcerated hemangiomas tended to be treated by conventional wound care and pulsed‐dye laser (OR, 2.03; 95% CI, 1.19–3.46; *P* < 0.0091), and non‐ulcerated hemangiomas were treated by topical glucocorticoid administration (OR, 2.57; 95% CI, 1.49–4.43; *P* < 0.0007) and surgical resection (OR, 2.04; 95% CI, 1.08–3.86; *P* < 0.0286).

However, propranolol has recently been suggested to be effective regardless of the presence or absence of ulcer formation, and as it has few adverse effects, it is expected to become the first choice treatment in the future.

Treatments

1. Oral propranolol

Hermans *et al.* 172 treated 20 previously treated patients with ulcerated infantile hemangioma using propranolol and compared them with 36 patients treated without propranolol. The administration was initiated by hospitalization, and the dose was increased from 0.7–1.0 to 2.0–2.5 mg/kg per day in three divided doses at an interval of at least 3 days. The blood pressure, heart rate and blood sugar level were monitored during the initial administration period, and the administration was continued on an outpatient basis until the age of 1 year. The mean age at the beginning of propranolol administration was 3.5 months, and the mean duration of administration was 9.1 months. Not only the color and elevation of the lesion but also pain was reduced from early after the beginning of administration. The administration was concluded before the age of 1 year in 19 patients, and no recurrence of ulcer was noted in any of these patients except that some reactivation (enlargement) of hemangioma was observed after the discontinuation in four of these patients.

The mean time until complete cure of ulcer was 8.7 weeks, and those in whom the administration was initiated later (>3.5 months) tended to require a longer time until cure than those in whom the administration was initiated earlier (*P* = 0.025). Also, analysis using Student’s *t*‐test showed a significant difference in the time until disappearance of the tumor, which was 8.7 and 22.4 weeks (*t* = 2.6, d.f. = 38, *P* = 0.012; 95% CI, 3.2–24.2) in the treated and control groups, respectively. Temporary sleepiness/malaise was observed in six patients, irritability before falling asleep in two patients, coldness of the limbs in six patients, anorexia in two patients and gastrointestinal disorders (diarrhea, vomiting) in one patient, but no adverse event was noted in nine patients.

Vercellino *et al.*,^202^ who started the administration at 1 mg/kg per day and increased to 2 mg/kg per day, and Sadykov *et al.*,^203^ who started the administration at 2 mg/kg per day, also reported that propranolol was effective.

- Topical and/or systemic administration of antibiotics

Kim *et al.* 204 topically administrated antibiotics in 40 patients with ulcerated hemangioma and reported that the results were better in 37 patients (92.5%), worse in none and no change in three patients (7.5%). They also systemically administrated antibiotics in 26 patients and reported that the results were better in 24 patients (92.3%), worse in two patients (7.7%) and no change in zero patients.

Wananukul *et al.* 205 topically and/or systemically administrated antibiotics in 41 patients with ulcerated hemangioma and reported improvement in 19 patients (46%).

Pandey *et al.* 206 treated 608 patients showing ulcer formation with an ointment containing an antibiotic (mupirocin, sodium fusidate, sisomicin or metronidazole) combined with systemic administration of an antibiotic (amoxiclav at 20–40 mg/kg per day) in those with ulcers with an area of more than 10 cm^2^ and examined the effectiveness of treatment according to the time until cure. The time until cure was 32.63 ± 13.06 days in superficial lesions, 42.89 ± 19.89 days in mixed lesions and 57.03 ± 16.12 days in extensive lesions, with a mean of 40.09 ± 19.41 days in all lesions combined, showing significant differences among the three groups (*P* < 0.05). They also reported that the time until cure was significantly longer in larger (>10 cm^2^) than smaller ulcers (*P* < 0.05).

- Dressings

Kim *et al.* 204 treated 25 patients using dressings and reported that the results were better in 23 patients (92%) and no change in two patients (8%). Oranje *et al.* 207 applied polyurethane film and reported rapid relief of pain and cure of ulcer in 1–2 months. In addition, Bauland *et al.* 208 treated 41 patients using a non‐adhering dressing containing an antibiotic and reported that the results were good in 26 patients (63.4%), moderate in five patients (12.2%) and little change in 10 patients (24.4%).

- Laser therapy

In the 1980s–1990s, there were reports^209^, ^210^ of argon, Nd:YAG and potassium titanyl phosphate laser, but recent reports^211^, ^212^ are primarily about treatment using dye laser. Morelli *et al.* 209 treated 37 patients with ulcerated hemangioma by dye laser irradiation (SPTL1b^®^ [Candela Corporation, Wyland, MA, USA]; wavelength, 585 nm; spot size, 5–7 mm; irradiation power, 5–6.8 J/cm^2^; pulse width, 0.45 ms) and reported that the number of irradiations until cure was one in 26 patients (68%) and two in eight patients (21%), and that the mean period from the first treatment until cure of ulcer was 2.84 ± 0.22 weeks. Lacour *et al.* 210 irradiated eight patients with ulcerated hemangioma that resisted conventional treatments using the same equipment and reported acceleration of cure. David *et al.* 211 performed dye laser irradiation (PhotoGenica V^®^ [Cynosure, Westford, MA, USA]; wavelength, 585 nm; spot size, 5–7 mm; irradiation power, 5–6.8 J/cm^2^; pulse width, 0.3–0.5 ms) in 78 patients and reported the effectiveness of laser therapy alone in 72 (92.3%). Also, Michel^212^ performed one or two irradiations using Dermobeam 2000^®^ (Deka MELA, Calenzano, Italy) with a cooling system 595 nm (two pulsed irradiations with a 10% overlap; spot size, 7 mm; irradiation power, 4–8 J/cm^2^) and reported resolution of pain in 10 of the 12 patients. Moreover, Di Maio *et al.* 213 performed laser treatment in 65 patients with hemangioma with ulcer and reported that the effect was excellent and that no clear adverse events were observed, because scarring, which was noted in a few patients, did not differ markedly compared with scarring that occurs after conventional treatments.

However, Kim *et al.* 204 treated 22 patients with pulsed‐dye laser and reported that the results were better in 11 patients (50%), worse in one patient (4.5%) and no change in four patients (18.2%), but warned that five patients in the proliferating phase showed ulcer formation after irradiation.

As observed above, although there have been several reports of the effectiveness of laser therapy against ulcer as factors of “benefit”, many reports are relatively old and lack controls, and the evidence is not considered sufficient. Further accumulation of cases is necessary. Laser may be effective in limited patients, but as there is the risk of ulcer formation as an adverse effect of laser irradiation of infantile non‐ulcerated hemangioma, greater caution is needed in treating already ulcerated lesions.

- Steroids

There have been few reports on steroid therapy focusing on ulcer. Kim *et al.* 204 treated seven patients by local steroid injections and reported that the results were better in four patients (57.1%), worse in one patient (14.3%) and no change in one patient (14.3%). They also systemically administrated steroid to 22 patients and reported that the results were better in 16 patients (72.7%), worse in one patient (4.5%) and no change in five patients (22.7%). Based on these results, they considered that the treatment was effective for reducing the lesion size, and there are few other reports suggesting the effectiveness of steroid. Considering that the patients are infants and that there are other treatment options, steroid cannot be recommended at present.

- Topical preparations of recombinant human platelet‐derived growth factor

Becaplermin 0.01% (Regranex^®^, Ortho‐McNeil Pharmaceutical, Raritan, NJ, USA) is a preparation for diabetic foot ulcer approved by the US Food and Drug Administration in 1997. Sugarman *et al.* 214 and Metz *et al.* 215 reported its effectiveness for the treatment of ulcerated hemangioma in one and eight patients, respectively, but its effectiveness cannot be appraised at present because of the small number of cases.

#### CQ16: Is intralesional corticosteroid injection more effective than systemic administration for infantile hemangioma?

*Recommendation*: Treatment using corticosteroid is effective for inducing early regression of hemangioma. While no significant difference is observed in the effectiveness between intralesional injection and systemic administration, attention to complications including those at the administration site, such as the periocular region, on local injection and those, such as hypertension and growth retardation, on systemic administration is necessary.

*Strength of recommendation*: 2 (weak).

*Evidence*: B (moderate).

*Comments*: As a result of primary screening, 99, nine and 35 papers were extracted from PubMed, Cochrane and JCRM, respectively, and four papers in English were subjected to secondary screening for this CQ. There was one report of an RCT, but the other reports were about case series while they evaluated a large number of cases. In addition, two papers on complications considered important in relation to intralesional corticosteroid injection for periocular lesions were added by manual search. Because there is a report of an RCT, and because other case series studies with a large number of subjects presented the results that there was no significant difference in the effectiveness of corticosteroid depending on the administration method, the strength of evidence was rated as B.

There was one report^216^ of an RCT focusing on “Is intralesional corticosteroid injection more effective than systemic administration for infantile hemangioma?”. In this trial, the subjects were divided into control, p.o. administration (prednisolone at 2 mg/kg per day every other day for 6 weeks) and intralesional injection (triamcinolone at 1–5 mg/kg with a maximum of 30 mg once a month for 6 months) groups, and the lesion size was significantly reduced in the treated groups compared with the control group. While no significant difference was noted between the p.o. administration and the intralesional injection groups, the reduction rate tended to be larger in the local injection group, and local injection was concluded to be slightly superior.^216^

There were reports^217^, ^218^ of case series with more than 1000 subjects, but the findings were not statistically analyzed. Although both intralesional injection and p.o. administration were effective, there was also a mixed group of intralesional injection and p.o. administration, the condition of patients varied among the three groups (intralesional injection, p.o. administration, mixed), and the effectiveness according to the administration method was not shown. Regarding complications, systemic symptoms, such as hypertension, retarded bodyweight gains and cushingoid appearance, were reported to be more frequent on p.o. administration than intralesional injection.^217^, ^218^ Moreover, concerning complications, in one report,^219^ periocular lesions were excluded from the targets of intralesional injection to avoid its effect on visual function. Indeed, there have also been case reports^220^, ^221^ that visual impairment was caused by occlusion of the retinal artery after intralesional corticosteroid injection for periocular hemangiomas. Currently, in Japan, intralesional corticosteroid injection is a treatment unapproved by the national health insurance system.

#### CQ17: Is topical therapy effective for infantile hemangioma?

*Recommendation*: Although it must be noted that there are no reports of comparison with placebo and that the degree of improvement is smaller compared with systemically administrated drugs, topical medication can be an option for the treatment of infantile hemangioma with no risk of complications if drugs with milder adverse effects are selected.

*Strength of recommendation*: 2 (weak).

*Evidence*: C (weak).

*Comments*: As a result of published work searches, a total of 111 papers consisting of 70, seven and 34 papers from PubMed, Cochrane and JCRM, respectively, were extracted. They included one RCT study. Including this RCT, 48 papers were extracted by secondary screening. In addition to the papers selected by secondary screening as closely related to the CQ, a total of 47 papers obtained by manual search were adopted as reference for the preparation of guidelines. There was one RCT, and comparative studies of therapeutic results by topical therapy and case series studies with a relatively large number of subjects were adopted as papers of relatively high quality, the strength of evidence was rated as C (weak).

In the reports related to the CQ:

1. Drug type: The drugs were classified as imiquimod, timolol, propranolol, corticosteroid and others.^215^, ^222^, ^223^, ^224^, ^225^
2. Drug concentration and dosage form: Imiquimod was used as a 5% cream,^222^, ^226^, ^227^, ^228^, ^229^, ^230^, ^231^, ^232^, ^233^ timolol as 0.5% ophthalmic solution or gel,^222^, ^223^, ^232^, ^234^, ^235^, ^236^, ^237^, ^238^, ^239^, ^240^, ^241^, ^242^, ^243^ propranolol as 1% ointment,^181^, ^234^, ^241^, ^244^, ^245^ and corticosteroid were often used as ointments of agents ranked as relatively strong such as clobetasol propionate, halobetasol propionate and betamethasone dipropionate.^246^, ^247^
3. Methods for topical application: Frequent administration methods were once a day every other day for imiquimod, two times a day every day at 1–2 drops each time for timolol, two times a day every day for propranolol and two times a day every day for corticosteroid.
4. Methods for efficacy evaluation: Comparison of gross findings and photographs were adopted in all papers. The area was compared using photographs in one report.^227^ There was also a report^228^ of half‐side test for a control.
5. Adverse effects: No systemic adverse effect was reported, and most adverse effects were local. Imiquimod caused pain, flare and erosion relatively frequently.^231^, ^233^ Few local adverse effects were reported for timolol and propranolol.^232^, ^234^, ^241^, ^242^, ^245^ No local adverse effects were reported also by corticosteroid.^246^, ^247^
6. Relative advantages of drugs: Imiquimod has been reported to have usefulness comparable with that of topical beta‐blockers, but it is not considered superior in terms of adverse effects.^226^, ^228^, ^230^, ^232^

Corticosteroid was not shown to be superior in efficacy compared with beta‐blockers.

There was also one RCT study^181^ concerning the CQ, which is related to topical propranolol concerning drugs. In this RCT, 15 each of a total of 45 subjects were allotted to oral (propranolol at 2 mg/kg per day, two times a day), topical (1% propranolol water soluble ointment, applied two times a day) and local injection (1 mg/1 mL, 0.2 mL/1 cm in diameter, 1 mL/injection at the maximum, one time a week) groups. Ten patients (66.7%) in the topical group responded, but they were fewer than 13 (86.7%) in the oral group. The time until the appearance of the effect and time until complete cure were also longer in the topical group than in the oral group. Concerning complications, none were observed in the topical group, but one patient in the oral group showed unexplained syncope as an adverse effect and was excluded. While decreases in the heart rate and blood pressure were observed in three in the oral group, they did not necessitate interruption of the study. In the local injection group, eight (53.3%) responded, but three were lost due to pain. From these results, the study concluded that topical therapy is an option to be evaluated for patients with a risk of adverse reactions to oral medication. While there were no reports of comparison under the same conditions, comparative studies of therapeutic results by topical therapy and case series studies with a relatively large number were adopted as relatively high‐quality papers. In all these reports, topical therapy of beta‐blockers (propranolol, timolol) was effective to an extent with no serious complications.

Thus, topical therapy, particularly of beta‐blockers is considered generally useful, but there has not been a report of its comparison with placebo, and further accumulation of cases is necessary.

Research by comparison between dye laser treatment and topical beta‐blocker therapy is considered to be necessary.

#### CQ18: Is compression therapy effective for infantile hemangioma?

*Recommendation*: Although an appropriate compression method must be performed for individual patients, compression therapy may be regarded as an option on condition that the therapy is carried out by a skilled physician. Sufficient attention to skin abnormalities and local/neighboring growth disturbance due to the compression are needed.

*Strength of recommendation*: 2 (weak).

*Evidence*: D (very weak).

*Comments*: While 23, one and 14 papers were extracted from PubMed, Cochrane and JCRM, respectively, only three case reports remained to be reviewed as a result of primary and secondary screening. Thus, the evidence level is very low at D (very weak).

According to a case report of ulcerated infantile hemangiomas of the limbs by Kaplan *et al.*,^248^ the ulcers of most patients showed rapid improvements and cure within 2 weeks by compression therapy using the self‐adherent wrap Coban® (3M, St. Paul, MN, USA) combined with topical treatment with an antibiotic ointment (or early systemic antibiotic administration when secondary infection was apparent). They concluded that, compared with antibiotic ointment alone, its combination with compression therapy was more effective, and is a safe and easy treatment that promotes regression of hemangiomas.

Ochi *et al.* 249 reported 12 cases of infantile hemangioma (nine girls and three boys with a mean age of 8.4 months; sites of the lesion were limbs in six, head and neck in five and trunk in one). By treatment using elastic bandages (five patients), Presnet® (ALCARE, Tokyo, Japan) (four), supporter (one), or Elatex® (ALCARE, Tokyo, Japan) and cryotherapy (two), the hemangiomas disappeared or decreased in size in 11 of the 12 patients, with only one (head and neck) showing no improvement. The time until the disappearance of the lesion in the 11 responders was 2 months to 3 years (mean, 19.5 months), no complications associated with compression therapy were noted, and the authors recommended early initiation of compression therapy if the site of the lesion can be compressed.

Totsuka *et al.* 250 treated three girls with parotid gland hemangiomas (mean age, 4.3 months) by splinting using a resin plate and compression using a handmade cap. The mean duration of treatment was 13 months (8–16 months), and the patients were followed up until a mean age of 4.6 years (2–7 years), resulting in clinical and echographic disappearance of hemangioma in all three. Because infantile hemangiomas often regress spontaneously, it is impossible to conclude that they regressed due to compression therapy, but they reported the therapy to be safe and effective.

Thus, concerning factors related to “benefits” of compression therapy, there are reports that suggest the effectiveness of compression methods appropriate for sites (elastic bandages, Presnet, splinting with a resin plate). However, it must be noted that they are all old reports. Concerning factors related to “harm”, while compression is a relatively safe and simple method without reports of serious complications, the occurrence of dermatitis and growth disturbance at the site of compression or surrounding areas is considered possible. The recommendation level was set at 2D with consensus of the present guidelines preparation committee on condition that the treatment is performed carefully by a skilled physician in consideration of these points. The present guidelines do not exclude compression therapy, but it is necessary to consider oral propranolol, p.o. administration or local injection of steroid, and laser therapy first for infantile hemangiomas that need treatment.

#### CQ19: Is glucose transporter 1 (GLUT‐1) immunostaining useful for the diagnosis of infantile hemangioma?

*Recommendation*: Immunostaining for GLUT‐1 is positive in the proliferating, involuting and involuted phases, shows high sensitivity and specificity, and is useful for the diagnosis of infantile hemangiomas if the clinical diagnosis is difficult.

*Strength of recommendation*: 2 (weak).

*Evidence*: C (weak).

*Comments*: To evaluate whether GLUT‐1 immunostaining is useful for the diagnosis of infantile hemangiomas, the published work was searched first for the following key words: infantile OR juvenile AND hemangioma AND marker AND immunohistochemistry.

The search of JCRM resulted in 26 hits, but none of them performed analysis of GLUT‐1 or evaluated its usefulness by comparing infantile hemangioma with other hemangiomas/vascular malformations even if GLUT‐1 was analyzed. The search of PubMed resulted in 182 hits. From these papers, those that deserved detailed analysis were selected according to the following criteria:

1. Those in which GLUT‐1 immunostaining was performed for infantile hemangioma or other hemangiomas/vascular malformations.
2. Those that were retrospective epidemiological studies rather than reports of one case.

Fifteen research papers selected by these criteria were analyzed in detail.

In seven of these reports,^251^, ^252^, ^253^, ^254^, ^255^, ^256^, ^257^ infantile hemangiomas were stained using GLUT‐1 simultaneously with other hemangiomas/vascular malformations, and differences in positive/negative results were evaluated. Of all cases reported in the seven papers, GLUT‐1 was positive in 268 of the 273 cases of infantile hemangioma and negative in 244 of the 247 cases of lesions other than infantile hemangioma. There were also four papers^258^, ^259^, ^260^, ^261^ in which GLUT‐1 staining was performed for clinically typical infantile hemangiomas and hemangiomas that need to be differentiated from infantile hemangioma although they were not simultaneously stained in the same paper. When the four papers were combined, GLUT‐1 was positive in all eight cases of infantile hemangioma and negative in all 49 cases of non‐infantile hemangioma. When the above cases are totaled, GLUT‐1 was positive in 276 of the 281 cases of infantile hemangioma and negative in 293 of the 296 cases of non‐infantile hemangioma, and the sensitivity and specificity of GLUT‐1 positivity for infantile hemangioma were 98.2% and 99.0%, respectively.

The usefulness of GLUT‐1 staining has also been confirmed by re‐evaluation of cases that were initially examined by hematoxylin–eosin (HE) staining alone.^262^, ^263^, ^264^, ^265^ There have been four papers in which cases were re‐evaluated using GLUT‐1 staining, and one paper reported that the diagnosis was impossible by HE staining alone in 18% of the cases.^262^

#### CQ20: What gastrointestinal examinations are useful for children suspected to have blue rubber bleb nevus syndrome? When should the examinations be started?

*Recommendation*: It is recommended to start screening by examinations including blood tests and fecal occult blood test as early as possible. In children suspected to have gastrointestinal bleeding, the usefulness of endoscopic examination, red blood cell scintigraphy (technetium‐99*m *[^99m^Tc]‐labeled red blood cells), and single photon emission CT–CT (SPECT‐CT) has been reported for the identification of the source of bleeding. If no abnormality is detected by screening, and search for gastrointestinal lesions needs to be performed to diagnose this disease or evaluate the future risk of bleeding, there is no standard for its timing. Among the examinations that led to the detection of gastrointestinal lesions in past reports, CT and MRI can be performed with relatively mild invasion and from an early stage.

*Strength of recommendation*: 2 (weak).

*Evidence*: D (very weak).

*Comments*: Gastrointestinal lesions of blue rubber bleb nevus syndrome (bean syndrome) are observed in the entire digestive tract, but they frequently appear, particularly in the small intestine. Because it is an extremely rare disease, the published work is primarily case reports and reviews, and there have been no reports of clinical studies of many cases that are relevant for the CQ. Therefore, we investigated examinations that were useful for the detection of gastrointestinal lesions in reports, primarily of child cases. Lesions in the small intestine are difficult to observe by conventional endoscopy, but techniques such as double‐balloon endoscopy, capsule endoscopy, CT enterography, CT and MRI as well as upper and lower gastrointestinal endoscopy have been reported to be useful.^266^, ^267^, ^268^, ^269^, ^270^, ^271^, ^272^, ^273^, ^274^, ^275^, ^276^

As a result of database searching, 11 papers in English were adopted through primary and secondary screening. All papers selected by these screening processes were case reports or case series, and the strength of evidence is D (very weak).

There is no clear standard as to when the examinations should be initiated. However, neonates who developed gastrointestinal bleeding shortly after birth have been reported,^270^ and the earliest possible examinations are necessary if this disease is suspected. Invasive examinations are difficult to perform in small children, but blood tests (presence or absence of anemia or consumption coagulopathy) and fecal occult blood tests can be performed. If gastrointestinal bleeding is suspected, procedures such as endoscopy, particularly double‐balloon endoscopy and capsule endoscopy, ^99m^Tc‐labeled red blood cell scintigraphy and ^99m^Tc‐labeled red blood cell SPECT‐CT have been reported to be useful for the determination of the source of bleeding.^266^, ^268^, ^271^, ^275^

If no abnormality has been detected by screening tests, and if a search for gastrointestinal lesions needs to be performed non‐emergently to diagnose this disease or evaluate the future risk of bleeding, there is no standard for the timing, which may vary among facilities. Among the above examinations, CT and MRI can be performed earlier and with relatively milder invasion, and are worth attempting if this disease is suspected. The necessity of the other examinations for the gastrointestinal lesions mentioned above should be considered when the patient reaches the age that tolerates the examinations.

#### CQ21: How are limb overgrowths to be managed in vascular malformations and syndromes?

*Recommendation*: If leg‐length inequality is insignificant, shoe lift is recommended. As significant inequality causes gait disturbance complicated with scoliosis, surgical treatment aimed to arrest epiphyseal growth is performed in the growth period. Shortening of the femur or tibia may be performed as an additional treatment. Bone elongation of the intact side is considered effective for the correction of leg‐length inequality.

*Strength of recommendation*: 2 (weak).

*Evidence*: D (very weak).

*Comments*: As a result of published work searches, 40 papers in English and four papers in Japanese were retrieved by primary screening. Of these papers, 17 in English and four in Japanese were extracted by secondary screening. As for the control of overgrowth of limbs, measures against leg‐length inequality and soft tissue hypertrophy are separately discussed and regarded as effective, but these papers were all classified either as case reports or as general discussions. Therefore, the evidence level is rated as “very weak”, and the recommendation level as “weak”.

In vascular malformations, typical disorders with hypertrophy of the affected limbs are Klippel–Trenaunay syndrome and Parkes Weber syndrome, and most of the papers refer to the management of limb overgrowth due to vascular malformations were written about these disorders. The published work regarding lesions at different sites is commented on below.

*Lower limbs*: In most reports, treatment for the overgrowth of the lower limbs were aimed to prevent physical disorders caused by leg‐length inequality. Some reports particularly mentioned treatment for foot lesions.

1. Correction of leg‐length inequality

If the leg‐length inequality is less than 2 cm, the management of leg length difference and accompanying scoliosis is considered possible by the use of shoe lift.^277^, ^278^, ^279^, ^280^, ^281^ If the leg‐length inequality is 2 cm or more, significant gait disturbance, postural abnormalities and compensatory changes in the contralateral limb are likely to develop, and before consequent unphysiological gait leads to irreversible impairment, surgical treatment to correct the leg‐length inequality should be considered.^277^, ^278^, ^279^, ^280^, ^281^ Long‐leg radiography is useful for determining the best time for surgery,^282^ and the measurement of the leg length by long‐leg radiography or CT is reported to be effective.^278^ Surgical treatment reported in the papers are as follows:

*Treatment for overgrown limbs affected by vascular malformations*: Jacob *et al.* 277 performed epiphysiodesis in 41 patients with a leg‐length inequality of 2 cm or more among 252 patients with Klippel–Trenaunay syndrome and reported improvement in more than 90% of the patients. The effectiveness of this surgery is also confirmed by other review articles.^277^, ^278^, ^279^, ^280^, ^281^ The effectiveness of shortening of the femur and tibia was reported in the review by Capraro *et al.* 278 The fixation period is considered to be shortened as a whole by simultaneously performing femoral or tibial shortening in addition to epiphysiodesis. Redondo *et al.* 280 recommended endoscopic growth control of the epiphyseal plate in the distal end of the femur for patients with a leg‐length inequality of 2 cm or more. Capraro *et al.* 278 did not recommend growth control with the epiphyseal stapling because of the unpredictability of the results and high frequency of complications. The appropriate time for surgical intervention on affected limbs is reported to be around the age of 11 years.^280^

*Elongation of the intact leg*: Tanaka *et al.* 283 performed bone elongation of the intact limbs using an external fixator in adult patients with mild structural scoliosis and reported that the procedure was effective for correcting the leg‐length inequality and scoliosis. Jacob *et al.* 277 also recommended bone elongation of the intact limb using Ilizarov external fixation apparatus in their review.

*Popliteal vein ligation*: Servelle^284^ hypothesized that elongation of the affected limbs was due to a high venous pressure and performed ligation of the popliteal vein of the intact limb in 48 children, and they reported significant improvement in leg‐length inequality. However, there are also negative views, saying its effectiveness is uncertain.^278^

- Foot lesions

Redondo *et al.* 280 recommended resection of the toes (ray resection) and debulking for wearing shoes and cosmetic improvement. Gates *et al.* 285 notably reported that compared with ray resection, wound healing of the stumps was poor after major resection.

*Upper limbs*: Asymmetry due to hypertrophy of the upper limb less frequently causes impairment of activities of daily living than that of the lower limb. In one article, resection in patients with functional impairment due to marked finger deformities is reported,^282^ but articles reporting treatment for upper limb overgrowth are very few. While debulking has been reported to be advantageous from the cosmetic viewpoint,^279^ it has also been reported to induce or exacerbate edema of the affected limb,^284^ causing complications including cicatricial contracture, recurrence of the lesion and refractory ulcer,^278^ and sufficient caution is necessary.

#### CQ22: Is surgical resection effective for soft tissue/superficial LM?

*Recommendation*: Although surgical resection is an effective treatment, it should be performed after comprehensive evaluation of cosmetic aspects, prognosis, functional prognosis, resectability and possibility of recurrence/complications.

*Strength of recommendation*: 2 (weak).

*Evidence*: D (very weak).

*Comments*:

Process of preparation of recommendation

Surgical resection is one of the major treatment options performed for LM. Although LM can be cured by total resection, the objective of treatment is not necessarily total resection, because the disease is not malignant, and surgical resection is often carried out for cosmetic, functional and symptomatic improvements. Cosmetic problems are considered to be particularly serious if the lesion is located in superficial areas such as the body surface and soft tissue. However, surgical resection has been known to cause complications including hemorrhage, infection, deformation and nerve paralysis.

In evaluating whether resection is effective, the balance between its positive aspects and negative aspects, such as complications, is important. For soft tissue/superficial LM, in which cosmetic improvement is important, problems including in what situations resection can be performed, whether there are criteria for the selection of resection and, as there are differences in the incidence of complications, cure rate and recurrence rate depending on the circumstances, whether its indications should be evaluated under different conditions are unclear. Therefore, the CQ, “Is surgical resection effective for soft tissue/superficial LM effective?”, was formulated, and the current knowledge was summarized.

Published work search and screening

As a result of the published work search, 105 papers in Japanese and 348 papers in English were subjected to primary screening. Of these papers, five in Japanese and 42 in English were subjected to secondary screening concerning this CQ. They did not include papers with a high level of evidence, such as an SR and RCT, and all of them were case series or case reports. As a result, in the evaluation of this CQ, the results and discussion in each case series were integrated.

Review of observational studies (case series)

The effectiveness of resection of LM was evaluated from the following five viewpoints: (i) effectiveness regarding the prognosis (mortality); (ii) resection rate of the lesions (resectability); (iii) functional outcome after resection (function); (iv) recurrence rate (recurrence); and (v) complications.

*Results of review*: Generally, the rate of successful surgical resection is high, and 90% or more resection is reported to be possible in 60% or more of the patients.^286^, ^287^, ^288^ This also applies to the head and neck region, which is the frequent site of the lesion.^286^ However, the percentage of resectable lesions decreases from the cystic to mixed and to cavernous type.^286^ Because many LM are distributed diffusely in the skin and subcutaneous adipose tissue and around structures including muscles, blood vessels and nerves, resection of the lesion involves resection of normal tissues in varying degrees. In lesions that show complicated distribution in the head and neck region, complications after surgical resection are observed relatively frequently. Serious complications including nerve paralysis, hematoma, local necrosis, sepsis, deformation, salivary fistula, hoarseness, airway obstruction and malocclusion have been reported,^286^, ^287^, ^289^, ^290^, ^291^, ^292^, ^293^, ^294^, ^295^, ^296^, ^297^ and facial nerve paralysis is likely to result from resection, particularly of LM infiltrating the parotid region.^289^ By the site, the incidence of complications increases as the area of involvement widens from unilateral to bilateral, from below to above the lingual bone, both sides, and both above and below the lingual bone.^293^, ^296^ Postoperative death may occur in patients with a severe neck lesion, but the extent of the effect of surgical resection is unclear.^287^, ^288^, ^298^ Postoperative recurrence is closely related to the resectability of the lesion depending on its distribution, and lesions that are difficult to resect due to a wide area of involvement and a strong tendency of infiltration have been reported to be associated with recurrence.^296^

*Limitations*: Indications for surgical resection vary among papers, and differences in the patient background must be considered in the evaluation of the effectiveness of resection. While there were many reports that surgical resection was performed in combination with sclerotherapy, and resection is considered to have been performed when more favorable results were expected from resection rather than sclerotherapy, criteria for their selection are unclear. Therefore, there is certainly the large bias of individual variation in the circumstances, and it was clearly impossible to conclude that resection is uniformly effective.

## Summary

While the effectiveness of surgical resection for soft tissue/superficial LM was evaluated, there was no published work with a high level of evidence. One of the major reasons is the diversity of the lesion type (cystic or cavernous), area of involvement and history of other treatments. Because of this diversity, the condition of patients is considered to show an extremely wide variation, and their generalization is impossible. However, if conditions, such as the type of the lesion (cystic or cavernous), site of origin and relationship with other treatments are restricted, tendencies were observed in functional prognosis, recurrence rate, and contents and incidence of complications.

While the resection rate of lesions by surgical treatment was suggested to be generally high, selection criteria for resection were unclear. Therefore, it is speculated that resection was performed for patients clinically judged to be treated more effectively by surgery. However, because there were some serious complications of surgical resection that persist as sequelae, their possibility should be evaluated carefully in performing surgical resection. The risk of resection has been suggested to vary with conditions of the lesion. The functional outcome is poor, and the recurrence rate and incidence of complications after resection are high, in those that occupy a wide area and those that are accompanied by symptoms such as airway obstruction.

From these observations, we propose at present, “While surgical resection is often effective, it must be selected in consideration of cosmetic aspects, prognosis, functional prognosis, resectability, and possibility of recurrence and complications”, despite limited scientific grounds. If complete resection of the lesion is possible, surgical resection may be performed as the first‐line treatment, but the possibility of other treatments including sclerotherapy in particular, should be evaluated according to the diverse conditions of individual patients, and surgical resection should be performed when other treatments are ineffective or when surgical resection is considered clearly superior.

#### CQ23: What is the optimal timing of surgery for soft tissue/superficial LM?

*Recommendation*: It is impossible to recommend optimal timing of surgery, and judgments according to the condition of each case are necessary.

*Strength of recommendation*: 2 (weak).

*Evidence*: D (very weak).

*Comments*:

Process of preparation of recommendation

Soft tissue/superficial LM are not malignant lesions. Emergent treatment may be necessitated by life‐threatening symptoms, such as airway obstruction, but the initiation of treatment immediately after the diagnosis is generally considered unnecessary. The natural course of the disease differs considerably among individuals, particularly in infancy, and the lesions may show a tendency of spontaneous regression but may also cause various functional problems due to rapid enlargement. Moreover, there are cosmetic problems characteristic of this disease in addition to functional problems, and early therapeutic effects are necessary to make social life comfortable. For these reasons, the selection of optimal timing of treatment, surgery in particular, is a major issue.

For the selection of the timing of surgical resection, conditions to obtain the best results as well as indications for resection must be evaluated, and sufficient consideration of the balance between merits and demerits depending on the timing of resection is necessary. Therefore, in this CQ, we attempted to summarize the presently available knowledge about “What is the optimal timing of surgery for soft tissue/superficial lesions?”.

Published work search and screening

As a result of a published work search, 67 papers in Japanese and 231 papers in English were subjected to primary screening. Of these papers, five in Japanese and 42 in English were subjected to secondary screening for this CQ. They included none with a high level of evidence, such as an SR and RCT, and all papers were case series or case reports. Therefore, the results and discussion in each case series were integrated in the evaluation of this CQ.

Review of observational studies (case series)

Defining “the optimal timing of surgery” mentioned in the CQ as “the timing of surgery at which good results can be obtained”, we aimed to evaluate the timing of surgery at which resection is effective, problems, such as complications are few, and namely “the best results” can be obtained as a whole. Conditions must be evaluated on the basis of the timing in addition to the effectiveness of surgery, but objective judgments were considered difficult in this evaluation. However, as it was considered possible to obtain information about the age and time of surgery from the published work reviewed in the previous CQ concerning the effectiveness, papers that evaluated the age at surgery were searched.

*Results of review*: Despite a careful review of the published work by secondary screening, there was no paper that analyzed cases from the viewpoint of optimal timing of surgery. There was information concerning the age at surgery, but its appropriateness was not evaluated. Papers that mentioned the timing of surgery are shown below.

Concerning the timing (age) of surgery, unless the size of the lesion is small or there are symptoms that require urgent treatment, such as respiratory disturbance, it is recommended to delay surgery until the age of 3 years by expecting spontaneous regression or for the ease of identification of surrounding structures during surgery, ease of control of bleeding and less problems with postoperative management.^294^ There was also a paper^295^ that suggested the necessity of the determination of the time of surgery in consideration of problems that change with age including the priority of securing the airway and appropriate nutritional management in neonates with head and neck and giant lesions, control of hemorrhage and infection and measures to prevent dysarthria and dental problems in infants, and skeletal and cosmetic problems in school‐age children, although it did not mention the optimal timing of surgery.

However, there was no paper that positively recommended resection without considering the time after the diagnosis or grounds for such a recommendation.

## Summary

As a result of the published work search for evaluating the CQ, “What is the optimal timing of surgery for soft tissue/superficial LM?”, there were papers that mentioned the timing of surgery, but none of them objectively evaluated its appropriateness. Therefore, no suggestion about the appropriate timing of surgery was obtained from the published work available at present, but there were a few papers suggesting that the decision to perform surgery should be made carefully.

Similar to the previous CQ, soft tissue/superficial LM of which the background vary in individual cases, and it is difficult to uniformly evaluate the effectiveness of resection. In clinical practice, in addition to medical reasons, social reasons including school attendance are considered to largely influence the decision of the time of resection. The results of RCT are necessary to obtain objective data, but it is practically very difficult to arrange an RCT fulfilling the above conditions.

While this CQ is a very important issue for patients and families as well as clinicians, there has not been objective evaluation of the optimal timing of surgery in the past. Presently, as quick decisions to perform surgery should be avoided, this guideline proposes, “The optimal timing of surgery cannot be decided in general, and judgments according to the condition of each case are necessary”.

#### CQ24: Is sclerotherapy effective for facial microcystic LM?

*Recommendation*: A wide range of drugs are used for sclerotherapy. Although comparison among drugs has not been made, and consensus regarding the methods or frequency of their administration has not been reached, improvements are observed after sclerotherapy in various symptomatic, functional and cosmetic (esthetic) aspects. However, complications including functional impairment have also been reported.

*Strength of recommendation*: 2 (weak).

*Evidence*: D (very weak).

*Comments*:

Process of preparation of recommendation

Published work search and screening

Concerning this CQ, 35 papers in Japanese and 92 papers in English (60 from PubMed, 32 from Cochrane) were retrieved. After their primary screening, six in Japanese and 18 in English were subjected to secondary screening concerning this CQ. Although they included three RCT, many of the other papers were case series or case reports. Therefore, in the evaluation of the draft recommendation concerning this CQ, the results and discussion in each RCT and case series were integrated. While the evidence is scarce, the papers judged to be useful for the preparation of the draft recommendation are presented as review data.

Review of case series

As a result of published work screening, it was found that the effectiveness of sclerotherapy for facial microcystic LM has been evaluated from the following viewpoints:

1. Treatment responses
  - Size
  - Symptoms
  - Functions
  - Cosmetics
2. Complications

The contents of the accounts concerning the effectiveness of sclerotherapy are summarized according to these viewpoints.

However, there were few reports that exclusively analyzed facial (and microcystic) LM, and lesions of the neck and other regions as well as the face were evaluated or different types of LM, such as cystic and mixed types, were reported together. In addition, the definition of the cavernous type and standard procedure of sclerotherapy (method and number of administrations) varied among reports, and these differences in background should be considered in the evaluation of the effectiveness of sclerotherapy.

The sclerosing agents used for the published work search ranged widely from OK‐432, bleomycin, ethanol, doxycycline to STS. However, as none of the papers reviewed for the preparation of these guidelines evaluated differences in the effectiveness for facial microcystic lesions among drugs or the method or number of administrations of each drug, these evaluation items were excluded in discussing this CQ.

1. Responses

*A. Size*: Many of the papers that referred to the regression rate of the lesion classified the responses into (i) excellent or complete (regression rate ≥90%); (ii) good or substantial (regression rate ≥50% and <90%); (iii) fair or intermediate (regression rate ≥20% and <50%); and (iv) poor or none (regression rate <20%).

Although there was no paper that collected cases of facial lesions alone, Yang *et al.* 299 reported that the regression rate after sclerotherapy was 90% or more in 19 (63%) of the 30 patients with head and neck lesions and 50% or more in 10 (33%). In addition, the regression rate was reported to be 50% or more in 18 (85.7%) of the 21 patients with head and neck lesions by Alomari *et al.*,^300^ and in 30 of the 31 patients to be 50% or more, who included those with mixed type lesions, by Chaudry *et al.* 301

Smith *et al.* 302 reported that none showed a response (complete or substantial) in 17 patients who underwent sclerotherapy, some of whom had mediastinal lesions. Giguere *et al.* 303 also reported that all five patients with head and neck lesions showed no response (poor) to the therapy. While these studies were RCT evaluating the time of sclerotherapy, the results suggest that sclerotherapy is not effective for microcystic lesions regardless of the time of treatment.

There was no paper that compared sclerotherapy and resection for facial microcystic LM.

*B. Symptoms*: There is no published work that evaluated this item based on objective data, and few reports referred to symptoms themselves. The information was limited to the report by Chaudry *et al.* 301 that symptoms disappeared after sclerotherapy using bleomycin in 75% of the patients who complained of pain and a few case reports^304^, ^305^ that symptoms, such as hemorrhage and respiratory impairment, were relieved after sclerotherapy.

*C. Functions*: Ravindranathan *et al.* 306 performed sclerotherapy in three patients with diffuse microcystic lesions extending from the face to the tongue and pharynx and reported that respiratory impairment and swallowing disorder due to airway obstruction observed before treatment were mitigated. Poonyathalang *et al.* 307 administrated STS to a patient with orbital lesions primarily complaining of visual defect and reduced visual acuity due to retrobulbar hemorrhage and reported alleviation of the symptoms, but relevant published work similar to that concerning symptoms was scarce.

*D. Cosmetic aspects*: Cosmetic improvements are difficult to evaluate objectively. Poonyathalang *et al.* 307 administrated STS to three patients with orbital lesions with exophthalmos as the primary symptom and reported improvement by measuring the degree of protrusion before and after the treatment. There have also been reports of objective assessment based on the degree of satisfaction in the patients’ families. According to Chaudry *et al.*,^301^ all patients with head and neck lesions (nine with microcystic lesions, 22 with mixed lesions) and their families reported improvements in the size and appearance of the lesions. In addition, Alomari *et al.* 300 treated 32 patients with mostly microcystic but including some cystic LM of the head and neck region by sclerotherapy and reported improvements compared with the condition before treatment by the families of 26 patients (81.3%).

- Complications

As complications in the facial region, there are a large number of reports^299^, ^301^, ^307^, ^308^, ^309^, ^310^, ^311^, ^312^ of transient complications associated with sclerotherapy, such as fever, local swelling and pain, intracystic hemorrhage and infection, although the lesions were poorly characterized in some reports. In addition, complications considered to have been caused by the effect of treatment, such as ulcer of the oral mucosa and tongue, facial nerve paralysis, leakage of saliva and respiratory insufficiency due to airway obstruction, have been occasionally reported.^303^, ^306^, ^307^ There have also been reports^307^, ^313^, ^314^ of an elevation of the intraorbital pressure, exophthalmos, intraorbital hemorrhage, corneal damage and external ocular muscle paralysis due to enlargement of the mass after sclerotherapy for ocular LM. There was also no published work showing the incidence of complications in facial microcystic LM.

As complications caused by sclerosing agents, skin ulcer and necrosis and nerve damage due to ethanol leakage, hypotension during anhydrous ethanol injection and epidermal detachment due to doxycycline have been reported.^300^, ^315^ However, there was no report of serious complications due to OK‐432. Pulmonary fibrosis is widely known to be a complication of bleomycin, but, according to Chaudry *et al.* 301 and Yang *et al.*,^299^ impairment of respiratory function does not occur at a dose routinely employed for sclerotherapy.

## Summary

In evaluating the CQ, “Is sclerotherapy effective for facial microcystic LM?”, analysis was performed from the viewpoints of responses to the treatment in terms of symptoms, functions and cosmetic (esthetic) aspects and complications, but few papers with a high level of evidence were found. While the degree of regression of the lesions by sclerotherapy varied widely, the size‐reducing effect of the therapy was consistently small unlike that in cystic lesions. Some papers referred to symptoms, functional outcome and cosmetic improvement, but they were insufficient for general discussion of sclerotherapy for facial microcystic LM. As complications characteristic of sclerotherapy, serious impairment may be caused by leakage of the sclerosing agent (ethanol in particular), and this point needs attention. Based on the above observations, it is difficult at present to evaluate indications for sclerotherapy against microcystic LM by formulating criteria. Therefore, for the future, it is considered necessary to evaluate the usefulness of sclerotherapy addressed by this CQ by designs such as RCT.

#### CQ25: Is sclerotherapy effective for intra‐abdominal LM?

*Recommendation*: Although there are many reports that sclerotherapy is useful, there is the risk of complications, and careful judgments about matters including the resectability of the lesion and selection of the sclerosing agent are necessary.

*Strength of recommendation*: 2 (weak).

*Evidence*: D (very weak).

*Comments*:

Process of preparation of recommendation

Lymphangioma is the most frequent lymphatic vessel disorder of the abdomen. Intra‐abdominal lesions are estimated to account for 10–20% of all LM, and the selection of treatment is difficult depending on the site of the lesion. While surgical resection is expected to be effective, less invasive treatments are considered necessary in view of stress to the patient and the possibility of severe complications such as lymphatic fluid leakage and bowel obstruction. Sclerotherapy, which is a major treatment for LM, is considered to be less invasive than surgery. Although positive therapeutic effects are expected, sclerotherapy is known to induce marked inflammation. Whether it can be performed safely without negative effects including complications and its long‐term effects are major clinical concerns. In addition, what therapeutic effects are expected or what complications should be anticipated after sclerotherapy for the intra‐abdominal lesion is also unclear. Therefore, the CQ, “Is sclerotherapy effective for intra‐abdominal LM?”, was formulated, and knowledge available at present was compiled.

Published work search and screening

As a result of the published work search, 19 papers in Japanese and 38 papers in English (32 from PubMed, six from Cochrane) were subjected to primary screening. Of these papers, two in Japanese and nine in English were subjected to secondary screening concerning this CQ. They included no papers with a high level of evidence, such as SR and RCT, and all were case series or case reports. Consequently, the results and discussion in each case series were integrated in the evaluation of this CQ.

Review of observational studies (case series)

The published work concerning the effectiveness of sclerotherapy for intra‐abdominal LM was reviewed from the viewpoints of: (i) therapeutic effects (decrease in lesion size, symptoms); and (ii) complications.

The drugs used for sclerotherapy ranged widely from OK‐432 to bleomycin, ethanol, doxycycline, STS, acetic acid, steroid/tetracycline and 50% glucose solution. According to our review, there was no paper that evaluated the differences in effectiveness of sclerotherapy in the abdomen according to the drug type or administration method or number of administrations of each drug.

*Results of review*:

1. Therapeutic effects

*A. Regression rate of the lesion*: Regression of lesions of intra‐abdominal LM by sclerotherapy was mentioned in five papers.^288^, ^316^, ^317^, ^318^, ^319^ According to the report by Chaudry *et al.*,^316^ the reduction rate was 90% or more in seven and 20% or more in one of the 10 patients with LM of the mesentery and retroperitoneum treated with doxycycline, and evaluation using imaging examination was not performed in two cases. The patient who showed a low regression rate had a mixed type of cystic and cavernous lymphangiomas, and the other patients had cystic lesions. Oliveira *et al.* 317 reported that the lesion regressed by 70% in one of the two patients with cystic lymphangiomas treated with OK‐432. Won *et al.* 318 reported one patient who showed complete disappearance of cystic retroperitoneal lesions after sclerotherapy using acetic acid. Shiels *et al.* 319 reported that cystic lesions responded to sclerotherapy using STS and ethanol in two patients, but there was no mention of the reduction rate. However, according to Alqahtani *et al.*,^288^ no effect was observed in 10 patients who underwent sclerotherapy using steroid/tetracycline or 50% glucose solution.

*B. Symptoms*: There were three papers that referred to symptoms of patients treated by sclerotherapy for intra‐abdominal LM.^316^, ^317^

According to Chaudry *et al.*,^316^ of the 10 patients who underwent sclerotherapy, three had chronic abdominal pain, three had acute abdominal pain, one had fever/chill, one had anemia and two had palpable masses, but the symptoms were alleviated by treatment in all patients, and no recurrence was noted.

Oliveira *et al.* 317 reported that sclerotherapy was performed in a patient with a palpable mass and in one with a palpable mass, abdominal compartment syndrome and a poor general condition. While the condition was alleviated in the patient who only showed a palpable mass after two courses of OK‐432 sclerotherapy, the treatment was changed to surgery in the patient who had abdominal compartment syndrome because of enlargement of the mass due to intracystic hemorrhage.

- Complications

Three papers specifically mentioned complications of sclerotherapy for intra‐abdominal LM. There was no report of deaths due to treatment‐related complications. Oliveira *et al.* 317 treated three patients by sclerotherapy using OK‐432 and reported that one of them developed sub‐bowel obstruction after the treatment and another required emergency surgery due to exacerbation of abdominal compartment syndrome induced by intracystic hemorrhage. Chaudry *et al.* 316 reported that doxycycline used for sclerotherapy leaked into the retroperitoneal space in one of the 10 patients but that the lesion regressed without any particular problem. Won *et al.* 318 performed sclerotherapy using acetic acid in one patient with retroperitoneal cystic lymphangioma. Although pain and hematuria were observed, they concluded that the relationship of hematuria with the therapy was unclear, because it was observed during menstruation.

*Limitations*: Sclerotherapy was often performed before, after or during surgical resection, and papers that reported the results of sclerotherapy alone were few. There was no paper that directly compared observation without treatment, sclerotherapy and surgical resection. Few papers analyzed intra‐abdominal lesions alone, and many papers included lesions in other areas or evaluated lesions in different intra‐abdominal regions including the mesentery, retroperitoneum and viscera collectively.

Moreover, differences in properties of LM, such as cystic, cavernous and mixed types, their definitions, criteria for the selection of sclerotherapy (combination with surgery, types of sclerosing agents and methods of their use, number of administrations) varied among papers, and few papers evaluated these matters separately.

Such differences in the patient background and contents of treatment must be considered in evaluating the effectiveness of sclerotherapy. In evaluating this CQ, particularly differences in morphology of LM and sclerosing agents were excluded.

## Summary

The CQ, “Is sclerotherapy effective for intra‐abdominal LM?” was evaluated from the viewpoints of therapeutic effect, symptoms/functions and complications, but no paper with a high level of evidence was found. While sufficient regression of the lesion and alleviation of symptoms were achieved by sclerotherapy in some patients, the response rate varied among reports, and information was insufficient for general discussion of sclerotherapy. Concerning treatment‐related complications, there have been reports of bowel obstruction associated with sclerotherapy, and attention to this condition as well as intracystic hemorrhage is considered necessary. However, there was no report of chylorrhea, which was reportedly caused by surgery.

Based on the above observations, it is presently difficult to determine indications for sclerotherapy in intra‐abdominal LM by setting up criteria, but as there was no published work that strongly ruled out intra‐abdominal LM as indications of sclerotherapy, these guidelines propose, “Although there are many reports that sclerotherapy is useful, there is the risk of complications, and careful judgments about matters including the resectability of the lesion and selection of the sclerosing agent are necessary”. For the future evaluation of this CQ, validation by a design with a high level of evidence, such as RCT, is considered necessary.

#### CQ26: Are patients with scarcely symptomatic intra‐abdominal LM candidates for treatment?

*Recommendation*: Because there is risk of treatment‐related complications, it is proposed to consider therapeutic intervention when the lesion tends to enlarge or has become symptomatic.

*Strength of recommendation*: 2 (weak).

*Evidence*: D (very weak).

*Comments*:

Process of preparation of recommendation

Intra‐abdominal LM occasionally presents with severe symptoms, such as abdominal pain, giant mass and bowel obstruction, but may also be asymptomatic and detected incidentally. Lesions may gradually enlarge and cause serious symptoms due to infection and intraluminal hemorrhage.

Under such circumstances, whether patients with nearly asymptomatic intra‐abdominal LM should be aggressively treated, or when intervention should be optimally performed during their long follow‐up period, are major problems that pose a clinical dilemma. Therefore, the CQ, “Are patients with scarcely symptomatic intra‐abdominal LM candidates for treatment?”, was formulated, and knowledge available at present was summarized.

Published work search and screening

As a result of the published work search, 206 papers in Japanese and 237 papers in English (230 from PubMed, seven from Cochrane) were subjected to primary screening. Of these papers, six in Japanese and nine in English were subjected to secondary screening concerning CQ26. They included no papers with a high level of evidence, such as an SR or RCT, and many of them were case series or case reports. Because seven papers among them described asymptomatic LM, their results and discussions were integrated to answer the CQ.

Review of observational studies (case series)

Seven papers^316^, ^317^, ^320^, ^321^, ^322^, ^323^, ^324^ among reviewed published work described asymptomatic LM. Fifteen cases reported in these papers were considered to have actually presented few symptoms (including asymptomatic patients who were incidentally detected by imaging studies to have intra‐abdominal masses at the sites as greater omentum, mesentery and retroperitoneum).

The published work was screened, and papers addressing issues concerning therapeutic intervention for scarcely symptomatic intra‐abdominal LM including, “What symptoms they may present with if they are left untreated?”, “By what studies and how often should they be examined?” and “What other treatments are available and how serious are complications or risk of each treatment?” were reviewed.

*Results of review*: From the published work reviewed, symptoms of intra‐abdominal LM (abdominal pain, bowel obstruction, torsion, infection, hemorrhage, vomiting/sucking difficulty, frequent urination and abdominal mass)^320^, ^321^, ^322^, ^323^, ^324^, ^325^, ^326^ are considered to be dependent on factors such as site, size and age. It is necessary to determine risk factors by stratification of these factors in the future.^320^, ^322^, ^325^

Reported complications in treated cases include recurrence that required re‐treatment,^321^ bowel obstruction,^317^, ^323^, ^324^ chylous ascites,^324^, ^326^ embolism,^317^ hemorrhage^317^ and wound infection. Embolism of the inferior vena cava after surgery^317^ and abdominal compartment syndrome after adhesion therapy^317^ were reported as severe complications. It deserves special attention that, if surgical resection is performed for mesenteric LM, the intestine may have to be resected with the lesion.^326^

While there have been reports^320^, ^322^ that intra‐abdominal LM with few clinical symptoms regressed during follow up, they may become symptomatic later (as observed in many case reports). For that reason, the opinion that intervention should not been chosen during the follow up until the lesion enlarges or new symptoms appear was frequently described.

*Limitations*: It should be noted that many asymptomatic cases may be left unreported and some asymptomatic lesions that had been detected were treated. There is no study with a high level of evidence indicating explicit criteria concerning the age, site or situation about whether intervention should be made for asymptomatic intra‐abdominal LM.

## Summary

The necessity of treatment of a patient with intra‐abdominal LM with few symptoms should be determined after evaluating the balance between the risk of treatment and non‐treatment considering its site and size as well as patient age. However, because research on indications for treatments has been insufficient so far and serious complications after treatment have been reported, deliberate evaluation for each patient is mandatory. When observation is selected, periodic imaging studies are recommended to optimize therapeutic intervention by detecting enlargement of the lesion. Also, if any symptom has developed during follow up, intervention should be considered. For these reasons, the recommendation, “Because there is risk of treatment‐related complications, it is proposed to consider therapeutic intervention when the lesion tends to enlarge or has become symptomatic” was adopted.

#### CQ27: What treatments are effective for refractory chylous ascites?

*Recommendation*: Conservative treatments, such as fasting, high‐calorie infusion and medium chain triglyceride (MCT), should be performed first, but, if they are ineffective, drug treatment, sclerotherapy and surgery may also be considered.

*Strength of recommendation*: 2 (weak).

*Evidence*: D (very weak).

*Comments*:

Process of preparation of recommendation

Refractory chylous ascites causes loss of large amounts of protein and lymphocytes, decreases in the blood lipid levels, and abdominal pain, unpleasantness and dyspnea due to abdominal distention and markedly reduces quality of life. The cause of ascites often remains unknown. Treatment of chylous ascites may require drainage to avoid abdominal distention. It is a very important point for clinicians to make proper judgments by understanding treatments and their effects and demerits. Therefore, it is considered beneficial to collect information about chylous ascites over a long period and compile guidelines. For this purpose, the presently available knowledge was collected by formulating the CQ, “What treatments are effective for refractory chylous ascites?”.

Published work search and screening

As a result of the search, 161 papers in Japanese and 728 papers in English (564 from PubMed, 164 from Cochrane) were subjected to primary screening. Of these papers, 15 in Japanese and 12 in English were subjected to secondary screening for CQ27. They included none with a high level of evidence, such as SR and RCT, and consisted of one multicenter and two single‐center case series and case reports. Consequently, we used the results and discussion of 27 papers judged for the preparation of the draft recommendation were integrated, although evidence was insufficient for the evaluation of this CQ.

Review of observational studies (case series)

As for causes of chylous ascites, congenital chylous ascites,^327^, ^328^, ^329^, ^330^, ^331^, ^332^, ^333^, ^334^, ^335^, ^336^, ^337^, ^338^, ^339^, ^340^, ^341^, ^342^ idiopathic chylous ascites,^328^ chylous ascites after laparotomy,^343^, ^344^, ^345^, ^346^ protein‐losing enteropathy,^345^ LM,^347^, ^348^ lymphangiectasis,^349^, ^350^ lymphangiomatosis^351^, ^352^ and lymphatic dysplasia^353^ were reported. None of the papers evaluated treatments according to the cause.

When treatments are categorized, conservative treatments (fasting, high‐calorie infusion, MCT), drug treatments, sclerotherapy and surgical treatment were performed.

*Results of review*: The results of review are presented below according to the treatment.

1. Conservative treatments

Whether the amount of ascites changes by fasting should be checked first.

High‐calorie infusion is often used with fasting, and because there was no report that ascites increased under the effect of high‐calorie infusion according to our review, it is recommended for nutritional support during fasting. In the multicenter case series reported by Bellini *et al.*,^327^ high‐calorie infusion/total parenteral nutrition was performed in 15 patients without adverse effects.

Medium chain triglyceride was used before, after and during treatment.^327^, ^328^, ^330^, ^331^, ^332^, ^333^, ^334^, ^335^, ^337^, ^339^, ^340^, ^341^, ^343^, ^345^, ^346^, ^348^, ^349^, ^350^, ^351^, ^352^ In the multicenter case series by Bellini *et al.*,^327^ MCT was reportedly performed in 14 patients without adverse effects.

- Drug treatments

In drug therapy for chylous ascites, primarily octreotide (a long‐acting somatostatin analog) was used, and no report that discussed the effectiveness of other drug therapies was found by the present published work search.

In the multicenter case series by Bellini *et al.*,^327^ octreotide was administrated to six of the 16 patients with chylous ascites for 8–38 days, and a decrease in chylous ascites was reported in all of them. In the single‐center case series by Huang *et al.*,^344^ two of the four patients with chylous ascites treated by high‐calorie infusion and octreotide administration were reported to have shown a decrease in ascites within 10 days. However, there has been a report^330^ that no effect was observed despite the administration of octreotide for 3 weeks. Concerning the dose of octreotide, it was administrated at 1 μg/kg per h,^327^ at 3 μg/kg per h,^332^ began to be administrated at 0.5 μg/kg per h and increased to 10 μg/kg per h by 1 μg/kg per h,^329^ administrated by continuous i.v. infusion at 0.5–2.0 μg/kg per h,^333^ and began to be administrated by s.c. injection at 2.5 μg/kg two times a day and increased every 2 days to 8 μg/kg two times a day.^330^ Regarding the time of the beginning of administration, the administration was started as no improvement was observed in chylous ascites after conservative treatments for 2 weeks,^330^, ^334^ and as chylous ascites was alleviated by conservative treatments but was exacerbated again.^333^ No adverse effects of octreotide administration were noted in the present review of the published work. Thus, no control study that evaluated the effect of octreotide on chylous ascites was found by the present published work search, and the level of evidence concerning the efficacy is low, but as there are case series and many case reports that chylous ascites was reduced by octreotide administration, it appears reasonable to consider drug treatment using octreotide for chylous ascites that does not respond to conservative treatments.

- Sclerotherapy

Sclerotherapy was performed in six patients in five case reports.^339^, ^347^, ^349^, ^351^, ^352^ The sclerosing agent was OK‐432 in five of the six patients and was Beta‐isodona® (Mundipharma GmbH, Cambridge, UK) solution in one.^349^ OK‐432 was locally injected into the lesion in four,^347^, ^351^, ^352^ administrated i.p. in one^352^ and administrated through the drain in two.^347^, ^352^ Concerning sclerotherapy, the number of reported cases that were reviewed was limited, and further accumulation of cases is considered necessary to establish its usefulness.

- Abdominal drainage, abdominal puncture and surgical treatment

Abdominal drainage and abdominal puncture are performed when organ compression symptoms (compartment syndrome and respiratory insufficiency) due to abdominal distention are present or possible, or when the drain is inserted postoperatively. However, drainage itself cannot improve chylous ascites, and treatments, such as infusion, blood preparations and blood transfusion, are necessary to supplement the ascites lost due to drainage.^327^, ^330^, ^331^, ^332^, ^333^, ^337^, ^338^, ^339^, ^340^, ^343^, ^345^, ^346^, ^347^, ^349^, ^351^, ^352^

Surgical treatment is reported to be frequently performed after conservative or drug treatments. According to the single‐center case series by Zeidan *et al.*,^343^ surgical treatment was performed in patients who responded poorly to conservative treatments for over a mean of 25.3 days. In other reports, surgical treatment was performed after conservative treatments for 1–3 months^328^, ^329^ and in patients with congenital chylous ascites 1–4 months after birth.^330^, ^334^, ^350^ Because it is often impossible to identify the leakage site of chylous ascites,^330^ there have been attempts to identify the leakage site by p.o. administration of a lipophilic dye (Sudan black, Sudan III) before operation.^328^, ^329^, ^336^, ^343^ When the leakage site can be identified, ligation, suturing, clipping and cauterization have been performed.^328^, ^334^, ^336^, ^343^, ^350^ In addition to reports of the usefulness of techniques to stop leakage, such as applying or sprinkling fibrin glue at the leakage site of chylous ascites or over the surrounding retroperitoneum,^329^, ^331^, ^343^, ^350^ and applying a patch of oxidized cellulose/resorbable local hemostatic agent,^331^, ^343^ there have also been reports of peritoneovenous shunting^349^, ^353^ and peritoneoamniotic shunting for fetal cases.^338^

There was no large clinical study in the past published work. Therefore, although the level of evidence is low, we consider that surgical treatment is recommendable for chylous ascites that does not respond to conservative or drug treatments, because it has been performed in case series and case reports for chylous ascites that did not respond to conservative or drug treatments for over approximately 1 month. Although techniques to enhance the response rate of surgical treatment, such as identifying the leakage site by using a lipophilic dye and applying fibrin glue or a patch of oxidized cellulose/resorbable local hemostatic agent, have been attempted, there are only case series and case reports, and none of the papers retrieved by the present published work search evaluated their usefulness.

*Limitations*: There was no published work that defined refractory chylous ascites based on the duration of illness or treatment responses. Therefore, we extracted and summarized factors that were considered to contribute to clinical refractoriness, such as the duration of illness and treatment responses, in each paper related to the treatment for chylous ascites. Also, as the cause of chylous ascites varies widely, the therapeutic effect is expected to differ depending on the cause, but no paper that was reviewed evaluated treatments according to the cause. Therefore, in the present evaluation, the statements are limited to treatments and their effects regardless of the cause.

## Summary

It was difficult to comprehensively discuss treatments, because its cause varied widely, and treatments for various causes were performed. Therefore, treatments were classified into conservative treatments (fasting, high‐calorie infusion, MCT), drug treatments (octreotide), sclerotherapy, abdominal drainage, abdominal puncture and surgical treatment, and the effects of each treatment were evaluated.

Treatments effective for refractory chylous ascites can be summarized as follows with the understanding that they may depend on the cause and that the level of evidence of the available reports concerning treatments and their effects is low. Conservative treatments, such as fasting, high‐calorie infusion and MCT, should be performed first because of the rareness of adverse effects. In patients who respond insufficiently to conservative treatments, drug treatments using octreotide can be considered as there have been case series and many case reports. Concerning sclerotherapy, the number of reported cases is small, and further large clinical studies will be needed to confirm its usefulness. Abdominal paracentesis and surgical treatments may be considered for chylous ascites that does not response to conservative or drug treatments for approximately 1 month.

Thus, the draft recommendation is “Conservative treatments, such as fasting, high‐calorie infusion and MCT, should be performed first, and, if they are ineffective, drug treatments, sclerotherapy and surgical treatments may be considered”. However, evaluation of this CQ by a design with a higher level of evidence, such as RCT, is considered necessary for the future.

#### CQ28: What kinds of complications are associated with treatments for intra‐abdominal LM?

*Recommendation*: Complications associated with sclerotherapy for intra‐abdominal LM include bowel obstruction, hemorrhage, pain, hematuria and chylous ascites. Surgical treatment of the disease can be associated with serious complications such as occlusion of the inferior vena cava and massive resection of the intestine as well as more common, wound infection, bowel obstruction, hemorrhage and chylous ascites.

*Strength of recommendation*: No recommendation.

*Evidence*: D (very weak).

*Comments*:

Process of preparation of recommendation

Patients with intra‐abdominal LM are treated with various modalities from non‐surgical therapy to surgical procedures. Treatment modality is selected depending on the patient’s state. Therefore, it is necessary for the clinician, patient and family to share information concerning complications that may be associated with treatments for implementing them smoothly. However, there are no resources that give a clear answer to this problem, and both clinicians and patients may become disconcerted. Therefore, the CQ “What kinds of complications are associated with treatments for intra‐abdominal LM?” was formulated, and information available at present was accumulated and integrated for the answer.

Published work search and screening

As a result of the published work search, 203 papers in Japanese and 602 papers in English (593 from PubMed, nine from Cochrane) were subjected to primary screening. Of these papers, 23 in Japanese and 27 in English were subjected to secondary screening concerning this CQ. They included no papers with a high level of evidence, such as SR or RCT, and all of them were case series or case reports. To answer CQ28, the results and discussion in each case series were integrated.

Review of observational studies (case series)

Complications in the CQ were evaluated by defining them as those encountered when patients with intra‐abdominal LM were treated, and reports on sclerotherapy and surgery were reviewed.

*Results of review*:

1. Complications associated with sclerotherapy

Sclerotherapy using OK‐432 was reported to be associated with bowel obstruction and hemorrhage for mesenteric LM,^317^ and chylous ascites for retroperitoneal LM.^326^ Sclerotherapy using acetic acid was reported to be associated with pain and hematuria in patients with retroperitoneal LM.^318^

- Complications associated with surgical procedures

Complete resection of both mesenteric and retroperitoneal LM by laparotomy was reported to be associated with wound infection^324^, ^354^ and bowel obstruction^323^, ^354^, ^355^ as common complications. There were reports of serious complications such as occlusion of the inferior vena cava^317^ and massive resection of the intestine was necessary due to diffuse infiltration of the LM tissue to the intestinal wall.^356^

In a report about complications associated with complete laparoscopic resection of intra‐abdominal LM by Tran *et al.*,^357^ resection was attempted in 47 patients, and conversion to laparotomy was necessary in three (6.4%) due to tight adhesion in two and intraoperative hemorrhage in one.

Partial resection by laparotomy was reported to be associated with persistent ascites over a long period that was refractory to the treatment.^355^

*Limitations*: Patients with intra‐abdominal LM are treated with various modalities including sclerotherapy and surgical procedures. Modalities were combined in many cases, and complications are often reported as those of entire treatment without more detailed information about those associated with individual treatment.

## Summary

For answering the CQ, “What kinds of complications are associated with treatments for intra‐abdominal LM?”, no published work with a high level of evidence was found, but foreseeable complications were listed from many case reports. Bowel obstruction, hemorrhage, pain, hematuria and chylous ascites were reported as complications of sclerotherapy. Serious conditions, such as occlusion of the inferior vena cava and massive resection of the intestine, as well as common complications, such as wound infection, bowel obstruction, hemorrhage and chylous ascites were reported as complications after surgical procedures.

Although the incidences and differences in complications in respect of the site and histological type are not shown in the published work, each patient with intra‐abdominal LM should be treated with sufficient evaluation of the site, size and symptoms. In addition, treatment must be implemented with sufficient understanding of the possible complications.

Thus, we propose “Complications associated with sclerotherapy for intra‐abdominal LM include bowel obstruction, hemorrhage, pain, hematuria and chylous ascites. Surgical treatment of the disease can be associated with serious complications such as occlusion of the inferior vena cava and massive resection of the intestine as well as more common, wound infection, bowel obstruction, hemorrhage and chylous ascites” as a recommendation draft.

#### CQ29: What treatments are effective for LM causing airway obstruction in the mediastinum?

*Recommendation*: Sclerotherapy is effective for macrocystic lesions, and surgical resection is effective for microcystic lesions. However, as the complication rate is relatively high, treatments should be selected according to the condition of each case.

*Strength of recommendation*: 2 (weak).

*Evidence*: D (very weak).

*Comments*:

Process of preparation of recommendation

Among LM, those that may cause airway obstruction due to their sites are life‐threatening. Lesions in the mediastinum cause respiratory disorders if they physically compress the trachea or bronchi and obstruct the airway or markedly protrude into the thoracic cavity and narrow it.

In such situations, aggressive and effective treatment is necessary, but the therapeutic approach must be selected carefully in consideration of the relationship of the lesion with the important organs around it such as the large cardiac vessels, mediastinal nerve and thoracic duct. However, the judgment is often difficult in clinical settings.

Therefore, the CQ, “What treatments are effective for LM causing airway obstruction in the mediastinum?” was formulated, and the presently available knowledge concerning matters including the risk of complications and prognosis of treatments, such as surgical resection and sclerotherapy, was summarized.

Published work search and screening

As a result of the published work search, 134 papers in Japanese and 227 in English (226 from PubMed, one from Cochrane) were subjected to primary screening. Of these papers, five in Japanese and 16 in English were subjected to secondary screening concerning this CQ. Because they included none with a high level of evidence, such as an SR or RCT, and all were case series or case reports, the results and discussion in each case series were integrated.

Review of observational studies (case series)

By screening of the published work, the following approaches were found for the treatment of LM in the mediastinum.

Therapeutic options are surgical resection, puncture and drainage, sclerotherapy (OK‐432, bleomycin, Ethiblock [Ethicon, Hamburg, Germany], anhydrous ethanol), drug treatments (Chinese herbal medicines such as *eppikajutsuto* and *ogikenchuto*) and no treatment. Of these approaches, surgical resection and sclerotherapy using OK‐432 have been evaluated in a relatively large number of cases, and reports of other therapy had an extremely limited number of cases, for example, reports of only one case.

*Results of review*: Boardman *et al.* 358 reported that, of the 97 patients with LM of the head and neck region, surgical treatments were necessary in six of the 12 patients with mediastinal lesions, that complications of surgery occurred in four of the six patients, and that long‐term nerve damage was observed in three of them. In addition, they reported that management by tracheotomy was necessary in 15% of all patients. Complete or nearly complete remission was observed in 92% of the patients, but they suggested that surgical treatments should be indicated only when there is airway obstruction or there is the risk of it, because surgical treatment of mediastinal lesions frequently causes complications.

Park *et al.* 359 reported that they surgically resected mediastinal LM in 12 patients. Seven of them had dyspnea and three were asymptomatic, but they were all judged to have indications for surgery due to symptoms or the tendency of the lesions to enlarge. A total of five recurrences were observed in four patients (33%) during a mean period of 3.6 years after the initial surgery, and all underwent by re‐resection. No perioperative death was observed, and, in a total of 25 cases including past cases, the overall survival was not different compared with that in healthy individuals over a follow‐up period of 11.5 years.

Smith *et al.* 302 performed local injection of OK‐432 in 16 patients with mediastinal LM and reported 60% or more regression of the lesion in 13 (81%). They also mentioned treatment responses according to the histological types and, by reporting responses (complete or nearly complete remission) in 94% of those with macrocystic lesions, 63% of those with mixed lesions, but 0% in those with microcystic lesions, suggested a macrocystic lesion to be a good indication for sclerotherapy using OK‐432. Although not from the viewpoint of airway obstruction, they reported that treatment using OK‐432 was more effective than surgical resection and less frequently caused serious complications.

*Limitations*: There have been no papers that directly analyzed treatments effective for mediastinal lesions expected to cause airway obstruction, and many papers reported cases of mediastinal lesions that responded to treatments. Therefore, we simply extracted matters relevant to this CQ from these reports.

## Summary

There was no published work with a high level of evidence concerning effective treatments for LM in the mediastinum causing airway obstruction. A few case reports that referred to the effects of surgery and sclerotherapy were observed, but it was difficult to present objective and specific figures concerning their effectiveness or safety. However, according to the available information, it should be noted that favorable responses have been obtained by OK‐432 local injection in macrocystic lesions and that complications due to surgical resection are likely to occur relatively frequently.

From these observations, we consider the following to be a therapeutic approach that can be proposed: “Sclerotherapy, such as that by local injection of OK‐432, should be considered for macrocystic lesions, and, for lesions that are technically difficult to treat by sclerotherapy or microcystic lesions, surgical resection should be considered with attention to complications. In addition, it is necessary to pay attention to the appearance of respiratory disturbances before and after these treatments and to constantly evaluate indications for securing the airway by intratracheal intubation or tracheostomy.” Therefore, at present, we recommend, “Sclerotherapy is effective for macrocystic lesions, and surgical resection is effective for microcystic lesions. However, as the complication rate is relatively high, treatments should be selected according to the condition of each case.”

#### CQ30: Should sclerotherapy be performed in infancy for a patient with head and neck LM affecting the airway?

*Recommendation*: In a patient with LM around the airway, there is risk of respiratory distress in infancy, while airway obstruction is likely to be exacerbated by sclerotherapy. Particularly when risk of airway obstruction is judged to be high or when the patient has already presented symptoms, it is proposed to perform sclerotherapy with sufficient preparations including airway management.

*Strength of recommendation*: 2 (weak).

*Evidence*: D (very weak).

*Comments*:

Process of preparing recommendation

Lymphangiomas of the neck, which are located in an exposed part of the body, may cause cosmetic problems that are important, but airway obstruction can be a particularly serious problem in some cases.

Sclerotherapy, which is one of the major treatment modalities, is most effective in patients with cystic LM, but swelling of the treated portion after the therapy may cause or exacerbate airway obstruction symptoms, especially in neonates. The upper airway will become less vulnerable to obstruction because it becomes less frail and wider as patients grow and respiratory distress tends to be unlikely. Therefore, it is occasionally difficult to determine how a patient who does not present any obstructive symptoms should be treated in infancy.

Thus, we evaluated this problem by formulating the CQ, “Should sclerotherapy be performed in infancy for a patient with the neck LM affecting airway?”.

Published work search and screening

As a result of search, 86 papers in Japanese and 135 papers in English (130 from PubMed, five from Cochrane) were subjected to primary screening. Of these papers, six in Japanese and 20 in English were subjected to secondary screening concerning this CQ. They included one SR, one RCT, two PS and one retrospective cohort study, but all the others were case series or case reports. Therefore, the results and discussion, primarily, in these SR, RCT, PS and retrospective cohort study, but also in other case series were integrated.

Review of observational studies

The published work concerning the effectiveness of sclerotherapy for head and neck LM in infancy was reviewed from the viewpoints of responses (prognosis [survival rate or mortality], size, symptoms and cosmetic improvement) and complications.

Sclerosing agents used as key words for the present published work search varied widely and included OK‐432, bleomycin, ethanol, doxycycline, STS and fibrin glue. No paper evaluated differences in effectiveness of various agents due to their methods of administration for lesions around the neck affecting the airway. Therefore, differences among agents were excluded from the evaluation of this CQ.

## *Results of review*:

1. Responses

*A. Prognosis (survival rate or mortality)*: According to the SR by Adams *et al.*,^360^ the mortality was 4.7% in 277 cases with head and neck LM. Because lesions around the airway were not the only target, and because sclerotherapy was not the only treatment modality, the paper has not quite rightly answered the CQ. However, because patients who died were all younger than 1 year of age and their causes of death are considered to have been mostly airway problems, such as airway obstruction and aspiration due to vocal cord paralysis in eight, and as at least one patient is judged to have died due to complications of invasive treatment, the paper is considered to indicate the risk of this disorder during infancy.

*B. Size*: Many of the papers that referred to the size regression evaluated it by four categories: (i) excellent or complete (≥90% regression); (ii) good or substantial (≥50% and <90% regression); (iii) fair or intermediate (≥20% and <50% regression); and (iv) poor or none (≤20% regression).

Ravindranathan *et al.* 306 treated five patients (aged 4–19 months) with cervicofacial LM by sclerotherapy using OK‐432 (in addition to Fibrovein® [STD Pharmaceutical Products Ltd, Hereford, UK] in two) and reported that the responses were good in one (20%) (cystic), partial in one (20%) (cavernous) and poor in three (60%) (two with cavernous lesions that required tracheotomy and one with cystic lesions in whom the condition improved to good after surgical resection). However, they did not mention the evaluation criteria for good, partial and poor.

According to the report of eight cases with head and neck LM by Leung *et al.*,^361^ all patients underwent sclerotherapy and 50% or more regression was observed in all patients, with complete regression in two. However, the patient age varied from 2 months to 11 years, and the types of LM were not mentioned.

Ogawa *et al.* 362 reported nine patients (including five preschoolers and toddlers, two school children and two adults) who underwent OK‐432 sclerotherapy for the neck LM and evaluated it to be markedly effective in eight, in whom the lesions mostly disappeared, and effective in one, who showed a 50% or more regression. Eight patients in whom the treatment was markedly effective consisted of one with mixed and seven with cystic lesions, and the one in whom the treatment was effective had a mixed type.

Cahill *et al.* 315 reported doxycycline sclerotherapy in 17 patients with head and neck LM (cystic in 10, mixed in seven [three required tracheotomy]), and its size regression was reported to be more than 90% in seven (41.2%) (cystic in six, mixed in one), 75–89% in four (23.5%) (cystic in two, mixed in two), 51–74% in four (23.5%) (cystic in one, mixed in three) and 25–50% in two (11.8%) (mixed in two).

Nehra *et al.* 311 reported doxycycline sclerotherapy in 11 patients with head and neck LM (cystic in seven, mixed in four; aged 2 days to 21 months) (later combined with surgical resection in three). The treatment results were excellent in five (45.5% of all patients) and satisfactory in two (18.2% of all patients) among seven patients with cystic lesions but poor in all four patients with mixed type lesions (36.4% of all patients). Particularly, three of the four patients with mixed type lesions required tracheal intubation shortly after birth and underwent sclerotherapy while intubated, but the effects were poor in all of them. Surgical resection was added in one and was under consideration in another.

*C. Symptoms*: According to Ravindranathan *et al.*,^306^ who reported five patients with cervicofacial LM (aged 4–19 months) treated with sclerotherapy using OK‐432 (in addition to Fibrovein in two), four exhibited symptoms of airway obstruction before treatment. Symptoms included dysphagia in two and dyspnea (including croup‐like symptoms) in four (some both). Symptoms were alleviated by sclerotherapy in two out of four (cystic one, cavernous one), but tracheotomy was necessary in the remaining two (cavernous in both) without improvement.

In the report of eight patients with head and neck LM and five patients with VM (aged 2 months to 11 years) by Leung *et al.*,^361^ their symptoms noted before treatment were mass or swelling (10 patients [77%]), pain after hemorrhage (two patients [15%]), skin discoloration (blue) (one patient [8%]), obstructive airway symptoms (six patients [46%]) and swallowing difficulty (one patient [8%]). All symptoms were alleviated by sclerotherapy (doxycycline for LM, STS foam for VM).

Arimoto *et al.* 363 reported a patient with cystic LM in the neck presenting 3 months after birth. The patient presented with respiratory distress at the age of 10 months due to enlargement of the LM following upper respiratory infection. While left vocal cord fixation due to the mass was confirmed by ultrasonography before treatment, aspiration of the cyst and steroid administration resulted in opening of the glottic area and regression of the mass with relief of wheezing and distress. Because they underwent sclerotherapy 2 months after the disappearance of symptoms, aspiration of internal fluid and steroid administration rather than sclerotherapy were directly effective for the alleviation of symptoms.

Kitagawa *et al.* 364 reported a patient with giant LM of the neck which had been prenatally diagnosed and was treated under *ex utero* intrapartum treatment by tracheal intubation after aspiration of the cyst. The lesion was reported to be refractive to subsequent sclerotherapy and tracheotomy was eventually needed.

Nehra *et al.* 311 reported that, among 11 patients with head and neck LM (cystic type in seven and mixed type of cystic + cavernous in four; aged 2 days to 21 months), three out of four with mixed LM presented respiratory distress soon after birth and were managed by intubation, but that all were extubated after sclerotherapy using doxycycline (1–3 times; median, 1.6 times).

*D. Cosmetic improvements*: No paper has reported cosmetic results in detail. Only sporadically they mentioned surgery for redundant skin after regression of cystic lesions by sclerotherapy.

- Complications

Complications associated with treatment for LM around the airway have been reported in many papers. They include temporary conditions caused by sclerotherapy such as fever,^292^, ^303^, ^312^, ^362^, ^365^, ^366^, ^367^, ^368^, ^369^, ^370^, ^371^ local swelling,^303^, ^312^, ^365^, ^367^, ^368^, ^370^, ^371^ pain,^288^, ^312^, ^362^, ^367^, ^370^, ^371^, ^372^ hemorrhage into the cyst^288^, ^303^, ^312^, ^368^ and infection.^288^, ^292^, ^303^, ^310^, ^312^, ^360^, ^365^, ^372^ The effects of treatment for head and neck lesions, such as respiratory distress due to airway obstruction^292^, ^303^, ^306^, ^312^, ^362^, ^365^, ^366^ as well as nerve palsy,^288^, ^292^, ^312^, ^360^, ^365^ have also been reported.

According to an SR about head and neck LM by Adams *et al.*,^360^ both nerve damage due to sclerotherapy and post‐therapeutic infection were reported in one (0.8%) out of 123 patients. Because nerve damage and infection after surgery were observed in 12 (10.2%) and seven (5.9%) out of 118 patients, respectively, the complication rate would be lower by sclerotherapy than by surgery.

Ogawa *et al.* 362 reported a patient aged 1 year and 5 months who developed airway edema after OK‐432 sclerotherapy for a cystic neck LM necessitating tracheal intubation for 3 days, and cautioned against sclerotherapy for LM around the airway in young children (particularly those <2 years old).

Kudo *et al.* 369 also reported two patients aged 11 months and 1 year and 11 months who were treated with OK‐432 sclerotherapy intubated in advance for fear of airway obstruction due to post‐therapeutic swelling. Tomemori *et al.* 373 also cautioned against sclerotherapy for LM in children aged less than 2 years as in the report by Ogawa *et al.* 362

On the other hand, Kudo *et al.* 369 reported two cases whose neck LM having been enlarged rapidly after suffering from measles or URTI. Arimoto *et al.* 363 also reported a patient with cystic LM of the neck 3 months after birth, who developed dyspnea due to enlargement of the lesion after URTI at 10 months and was about to be intubated.

Regarding complications due to sclerosing agents, Cahill *et al.* 315 reported those by doxycycline, STS and absolute ethanol. They reported delayed complications, such as Horner’s syndrome, transient left lip weakness, right facial nerve palsy and transient left hemidiaphragm paralysis, in addition to periprocedural complications such as hemolytic anemia after doxycycline injection in two patients, hypoglycemic and metabolic acidosis in three neonates, transient hypotension during absolute alcohol instillation and self‐limiting skin excoriation secondary to pericatheter leakage of doxycycline. Other reported complications include permanent vocal cord paralysis after local ethanol injection,^374^ serious complications after OK‐432 injection such as death due to pulmonary embolism,^375^ deaths due to pulmonary complications after treatment using bleomycin^376^, ^377^ and leukocytopenia due to bleomycin.^368^

*Limitations*: There are few papers that solely analyzed LM around the cervical airway. Most papers included lesions involving not only the neck but also the craniofacial and other parts of the body and reported LM with different properties such as cystic and mixed types. In addition, definition of cavernous lesions and methods of sclerotherapy (injection techniques, number of injections) were not similar among papers, and differences in these backgrounds must be taken into consideration to evaluate the effectiveness of sclerotherapy.

## Summary

The CQ, “Should sclerotherapy be performed in infancy for a patient with head and neck LM affecting the airway?”, was evaluated from the viewpoints of responses (prognosis [survival rate or mortality], decrease in size, symptoms, cosmetic improvements) and complications. Because there have been some reports on the risk of respiratory distress due to LM around the airway in infants, and therapeutic intervention is necessary even in infants when the risk is high or they have already developed symptoms. Such intervention is made by sclerotherapy or surgery, and as surgical resection is associated with the risk of more serious complications than sclerotherapy, intervention by less invasive sclerotherapy is recommended. Sclerotherapy is considered to be very effective because of the high regression rate of the lesion and symptom/function‐improving effect. However, its effect varies depending on the disease type, being somewhat less effective in the cavernous and mixed types than in the cystic type. Furthermore, when it is performed for the lesions around the airway, it may be associated with the risk of exacerbation of airway obstruction symptoms due to reactive enlargement of the lesion. Thus, we formulated the recommendation, “In LM around the airway, there is the risk of respiratory disturbances from infancy, but airway obstruction is likely to be exacerbated by sclerotherapy. Particularly when the risk of airway obstruction is judged to be high or when symptoms have appeared, it is proposed to perform sclerotherapy with sufficient preparations including securing the airway.”

#### CQ31: Is surgical resection effective for LM of the tongue?

*Recommendation*: Surgical resection is effective for reducing the size of the lesion and alleviating symptoms and functional impairment. However, total resection is often difficult, and careful decision‐making is required in consideration of the possibility of complications and recurrence.

*Strength of recommendation*: 2 (weak).

*Evidence*: D (very weak).

*Comments*:

Process of preparation of recommendation

While the tongue is one of the frequent sites of LM, the lesion is often distributed widely over the neck rather than localized in the tongue. LM of the tongue not only cause cosmetic problems, such as protrusion from the mouth and bleeding, but also readily occupy the oropharyngeal cavity and cause functional problems such as disorder of mouth closing, difficulty in speaking, respiratory disturbances and impairment of oral food intake. These conditions are treated at departments including plastic surgery, oral surgery, otorhinolaryngology and pediatric surgery. LM of the tongue are treated by surgical resection or sclerotherapy, but comprehensive evaluation of the condition of individual cases including the distribution of the lesion in the tongue, involvement of other areas and cyst components, and vascular distribution in addition to general information, such as the risk of complications and recurrence in each treatment, is necessary.

Therefore, the CQ, “Is surgical resection effective for LM of the tongue?” was formulated, and the present knowledge about the effectiveness of surgical resection of the lesion, particularly by partial glossectomy was summarized.

Published work search and screening

As a result of the published work search, 29 papers in Japanese and 76 papers in English (75 from PubMed, one from Cochrane) were subjected to primary screening. Of these papers, two in Japanese and 10 in English were subjected to secondary screening concerning this CQ. They included one retrospective cohort study, but most other papers were case series or case reports. Consequently, in the evaluation of this CQ, the results and discussion of the cohort study and each case series were integrated.

Review of observational studies (case series)

The effectiveness of surgical resection of LM of the tongue was evaluated from the viewpoints of resectability of the lesion, symptoms, function and cosmetic improvements as elements of responses as well as complications and recurrence.

## *Results of review*:

1. Responses

*A. Resectability of the lesion*: Twenty‐four cases of tongue lesions treated by surgical resection alone were reported in four papers. Catalfamo *et al.* 378 performed surgical resection of localized masses including normal structures with a margin of 1 cm in the horizontal direction and reported that the size of tongue lesions was reduced in eight (88.9%) of the nine patients.

Concerning large lesions impossible to resect totally, Boardman *et al.* 358 reported 13 cases of partial surgical resection, but several operations were often necessary to reduce lesion size. A total of two case have been reported,^379^, ^380^ and the lesion size was reduced in both. Although differences were observed in re‐enlargement after surgery, they are discussed in detail in “(2) Complications”.

In one case report, sclerotherapy was performed 15 times, but the lesion size could not be reduced, and surgical resection was performed, eventually resulting in a favorable outcome without recurrence.^381^

According to a report of 89 cases of head and neck LM by Lei *et al.*,^291^ the outcome was excellent in 73 (82%) and good in 16 (18%) although it was not a report of cases of tongue lesions alone. They included 43 cases of tongue lesions.

In addition, a few papers^382^, ^383^, ^384^, ^385^ that suggested the effectiveness of combinations of surgical resection with sclerotherapy and laser therapy were observed. Wiegand *et al.* 383 classified the disease into four stages according to the area of involvement and reported that the stage can be a prognostic factor. Surgery was effective, and complications were rare, when the lesion was localized in the superficial layer and part of the muscle layer. Surgical resection can also be effective, but complete resection is difficult, when the lesion extends over the entire muscle layer or to the tongue base and neck. Therefore, partial resection is often repeated and combined with laser therapy and sclerotherapy, but the recurrence is observed very frequently, and the results did not contradict the reports mentioned below in the section of the recurrence rate.^291^, ^358^

*B. Symptoms*: A wide variety of symptoms have been reported depending on the site of the mass, and they include tongue discomfort, bleeding, pain and difficulty in oral feeding.^386^ Roy *et al.* 387 reported that bleeding from the tongue surface, pain and eating difficulty were alleviated by cauterization.

*C. Functions*: In most patients who exhibited functional impairment, the lesions were so extended that they were no longer indications for one‐time surgical resection. Large masses located at sites such as the tongue base cause respiratory disturbances, swallowing disorders and difficulty in speech. According to the report by Azizkhan *et al.*,^385^ oral intake of normally cooked food became possible in 14, and normal vocalization became possible in eight, of the 21 patients with tongue base lesions. In addition, five of the 17 patients who needed tracheotomy were weaned.

*D. Cosmetic improvements*: Objective evaluation of cosmetic effects is also difficult.

Azizkhan *et al.* 385 reported that, of the 20 patients, excluding one with severe deformity who died, deformity of structures around the tongue, such as the mandible and maxilla, was mild in six, moderate in five and severe in nine. There have been a few reports that cosmetic improvements were also observed in patients who showed a reduction of the tongue size by surgical resection, but objective evaluation is insufficient.

- Complications

Although the properties of the lesions are unclear in some papers, facial nerve paralysis, vagus nerve paralysis, infection, hematoma, seroma, salivary leakage, ruptured suture and skin flap necrosis have been reported as complications of the facial region. There have also been reports of temporary complications such as pain and hemorrhage.

- Recurrence

There have been a few postoperative evaluations reporting that no reactivation that clinically required treatment was observed. Lei *et al.* 291 reported greater details: recurrence was observed in 21 (23.6%) of 89 patients and was more frequent in those aged less than 1 year, those with lesions in the oral cavity/face, those with lesions at three or more sites and those with microcystic lesions. According to Boardman *et al.*,^358^ LM of the tongue recurred in 12 (48%) of 28 patients, more often than other head and neck lesions. As factors related to this more frequent recurrence of lingual LM, more frequent involvement of other regions, such as the floor of the mouth, and a high percentage of microcystic lesions (70%) have been suggested. Of the two patients treated by surgical resection alone, one who underwent resection of the middle part of the tongue showed no re‐enlargement for 1 year or longer after surgery,^379^ but surgery was repeated three times in the one who underwent marginal resection.^380^ This patient who underwent repeated resections also showed no re‐enlargement, although the time of the last resection is unclear.

*Limitations*: In some papers, surgical resection was combined with other treatments,^381^, ^382^, ^383^, ^384^, ^385^, ^387^ lesions in other areas such as the neck were included^291^ and the lesion types were unknown. The lack of standardization of subjects and uniformity of the definition or time of recurrence must be considered in the evaluation of the effectiveness of surgical resection.

## Summary

Many papers suggest that surgical resection is effective for reducing the size of lingual LM. However, in patients with large lesions, lesions extending to structures other than the tongue, and microcystic lesions, several resections or combination of resection with other treatments such as sclerotherapy and laser therapy were necessary, and the recurrence rate tended to be higher. While a few papers referred to symptoms, functional outcome and cosmetic improvements, none showed a high level of evidence, and the evidence was insufficient for general discussion of the effectiveness of surgical resection.

Therefore, concerning the effectiveness of surgical resection for LM of the tongue, “Surgical resection is effective for reducing the size of the lesion and alleviating symptoms and functional impairment. However, total resection is often difficult depending on the distribution of the lesion, and careful decision‐making is required in consideration of the possibility of complications and recurrence”, was proposed as a draft recommendation.

#### CQ32: Is aggressive surgical intervention effective for chylous pleural effusion in the neonatal period?

*Recommendation*: For chylous pleural effusion refractory to conservative treatments, surgical procedures, such as pleurodesis, ligation of the thoracic duct and pleuroperitoneal shunting, may be effective.

*Strength of recommendation*: 2 (weak).

*Evidence*: D (very weak).

*Comments*:

Process of determining recommendation

Primary chylous pleural effusion during the neonatal period is often refractory and can be fatal. Thoracic drainage is performed for respiratory insufficiency due to accumulation of pleural effusion, followed by conservative treatments, such as nutritional therapy, steroid and octreotide therapy, conducted primarily by a neonatologist until resolution of chylous pleural effusion.

In refractory cases that do not respond to these conservative therapies, surgical intervention, such as ligation of the thoracic duct and pleurodesis, may be performed. However, no sufficient consensus has been obtained concerning their effects. To evaluate problems, such as at what point surgical intervention should be made and whether aggressive surgical intervention is effective for such a condition, the CQ, “Is aggressive surgical intervention effective for chylous pleural effusion in the neonatal period?”, was formulated, and the knowledge available at present was summarized.

Published work search and screening

As a result of the published work search, 98 papers in Japanese and 264 papers in English (262 from PubMed, two from Cochrane) were subjected to primary screening. Of these papers, eight in Japanese and nine in English were subjected to secondary screening concerning this CQ. They included none with a high level of evidence, such as an SR or RCT that evaluated surgical treatment, and all papers were case series or case reports. Consequently, the results and discussion in each of the case series judged to be useful for the preparation of the draft recommendation were integrated although they were weak as the evidence for the evaluation of this CQ.

Review of observational studies (case series)

The published work concerning the effectiveness of surgical treatment for chylous pleural effusion in the neonatal period was reviewed from the viewpoints of responses and complications.

## *Results of review*:

1. Responses

Surgical treatment for neonatal chylothorax is performed in patients who respond insufficiently even to thoracic drainage in addition to nutritional therapy using MCT milk or total parenteral nutrition or drug treatment such as octreotide administration.

The methods for surgical intervention found by the present published work review included ligation of the thoracic duct and pleuroperitoneal shunting as well as pleurodesis with OK‐432 administration, intrathoracic infusion of fibrin and povidone‐iodine administration, and some patients diagnosed *in utero* underwent pleuro‐amniotic shunting. Cases in which mildly invasive treatments, such as thoracoscopic ligation of the thoracic duct and intrathoracic fibrin application, have been reported in addition to those who underwent thoracic duct ligation by thoracotomy.

Treatments that were performed before surgery and their periods were not uniform. In addition, because there are cases that developed chylous pleural effusion after surgery and those of congenital chylothorax, the diversity of the patient background must be taken into consideration in the efficacy evaluation.

Among the surgically treated cases, those in whom chylous plural effusion disappeared, respiratory symptoms were alleviated and weaning from the respirator became possible have been reported.^388^, ^389^ In addition, the absence of recurrence or reactivation is considered to be a point.^388^, ^389^, ^390^, ^391^ There were reports that chylous pleural effusion after thoracic surgery was resolved by drainage alone. Cleveland *et al.* 392 considered conservative treatments, such as total parenteral nutrition, octreotide and diuretic administration, to be the best and, observing that, of the poor responders, the mortality was 80% in five who continued to be managed by conservative treatments but 0% in four who underwent additional surgery, reporting that surgical treatment contributed to the reduction of the mortality. According to the guidelines for the treatment of chylous thoracic effusion by Buttiker *et al.*,^393^ conservative treatment should be continuing for approximately 3 weeks but should be abandoned thereafter because of the risk of nutritional disturbance, and increased susceptibility to infection and liver disorders. However, Kaji *et al.* 394 reported that it is difficult to set a clear period of conservative therapy, because the effectiveness and success rate of surgical treatment are unclear.

- Complications

As complications due to sclerosing agents, fever and increased inflammatory reaction due to the administration of OK‐432 as well as pulmonary abscess and temporary flaccidity and protrusion of the upper abdominal region considered to have been due to intercostal nerve damage have been reported. While chyle leakage in the abdominal cavity was noted in a patient who underwent pleuroperitoneal shunting, there were no reports of fatal complications.

*Limitations*: Surgical treatment was performed in most reported cases when responses to conservative therapy were not obtained. Therefore, it must be assumed that the results of evaluation of this CQ are based on data concerning the effectiveness of surgery performed with conservative therapy.

## Summary

The published work was reviewed concerning the effectiveness of aggressive surgical intervention for neonatal chylous thoracic effusion from the viewpoints of responses and complications, but no objective study with a high level of evidence was found. In most reported cases, surgical treatment was performed when responses to conservative treatments were poor. Therefore, it is difficult to compare surgery with other therapies, and the evaluation of the period of conservative treatment before surgery remains insufficient. However, there was a paper that proposed surgical intervention after attempting conservative treatments for 3 weeks as a standard.

Thus, surgical intervention for neonatal chylous pleural effusion is characterized at present as an approach that may be effective but should be evaluated when the condition is not improved by other treatments, and “Surgical procedures, such as pleurodesis, ligation of the thoracic duct, and pleuroperitoneal shunting, may be effective for chylous pleural effusion refractory to conservative treatments” is proposed as a draft recommendation.

#### CQ33: What treatments are effective for refractory chylous pleural, and pericardial effusion and respiratory disturbances of the patients with generalized lymphatic anomaly (GLA) and Gorham–Stout disease (GSD)?

*Recommendation*: While treatments including surgery, sclerotherapy, radiotherapy, nutritional therapy and drug therapy are conducted, there is presently no effective treatment with a high level of evidence. Treatments should be selected in consideration of complications and adverse effects according to individual symptoms.

*Strength of recommendation*: 2 (weak).

*Evidence*: D (very weak).

*Comments*:

Process of preparation of recommendation

Generalized lymphatic anomaly and GSD are refractory diseases that cause a wide variety of symptoms in the entire body and are difficult to diagnose and treat. The investigation by the Health and Labour Sciences Research group (Ozeki group) carried out by 2013 showed that the mortality is particularly high when the patients had thoracic lesions.

Among the thoracic symptoms, chylous pleural effusion/pericardial effusion are often refractory and occasionally fatal. While information about the disease is extremely limited because of its rareness, case reports are being globally accumulated as chronic cases are managed on an outpatient basis, and as severe cases are treated intensively.

Presently, no radical treatment for these refractory diseases is known, but the CQ, “What treatments are effective for refractory chylous pleural, and pericardial effusion and respiratory disturbances of the patients with GLA and GSD?” was formulated to compile the knowledge about what treatments are effective as it is a problem of clinical importance.

Published work search and screening

As a result of the published work search, 208 papers in Japanese and 617 papers in English (598 from PubMed, 19 from Cochrane) were subjected to primary screening. Of these papers, two in Japanese and 25 in English were subjected to secondary screening concerning CQ37. They included no studies with a high level of evidence, such as an SR and RCT, and all were reports of 1–2 cases. Therefore, the evaluation of this CQ was performed by integrating the results and discussion in case series judged to be useful for the preparation of the draft recommendation despite the lack of evidence.

Review of observational studies (case series)

The effectiveness of various treatments for refractory GLA and GSD was evaluated according to the prognosis and the presence or absence of improvement in imaging findings, improvement in symptoms, improvement in airway obstruction, enlargement of the lesion, regression, treatment‐related complications, recurrence and reactivation.

*Conditions of patients*: The cause of chylous pleural and pericardial effusion is lymphorrhea from lymphatic vessel tissue lesions that have primarily invaded the mediastinum and pleura, and lymphorrhea from osteolytic lesions of the ribs and vertebrae was also observed. Respiratory disturbances were caused by pleural effusion, chylous pleural effusion, pericardial effusion, and direct invasion of the mediastinum and lungs.

*Results of review*: As surgical treatments for chylous pleural effusion, procedures, such as thoracentesis, thoracic drainage, ligation of the thoracic duct and pleural decortication, have been performed, and local lesions were surgically resected. In most cases, thoracentesis and thoracic drainage were performed, but chyle leakage was not resolved. As for complications, there were patients who developed hypovolemic shock and required blood transfusion and catecholamine administration or supplementation of albumin, immunoglobulin and clotting factors.^395^, ^396^, ^397^ While chylous pleural effusion was controlled in some patients who underwent ligation of the thoracic duct,^397^, ^398^, ^399^, ^400^, ^401^, ^402^, ^403^, ^404^, ^405^, ^406^, ^407^, ^408^ the treatment was performed in combination with other surgical procedure or radiotherapy in all cases.^400^, ^402^, ^408^ There was one case that showed improvement in respiratory disturbance.^406^ As complications of ligation of the thoracic duct, splenomegaly and lymphorrhea^405^ and left‐sided pleural effusion^397^, ^405^ have been reported. In the patients who showed marked improvements in chylous pleural effusion^395^, ^405^, ^408^ after pleural decortication,^395^, ^396^, ^401^, ^403^, ^404^, ^405^, ^408^, ^409^ the procedure was performed in combination with other surgical treatments or sclerotherapy, and there was no mention of complications. There were cases that showed marked improvements in chylous pleural effusion^396^, ^400^, ^405^, ^408^ among those who underwent surgical resection of local lesions including splenectomy,^396^, ^397^, ^400^, ^405^, ^408^, ^410^, ^411^, ^412^ but the procedure was performed in combination with other surgical treatments in most of them. Hemorrhage was reported as a complication.^410^ Among other treatments, pleuroperitoneal shunting^403^ and lung transplantation^413^ were performed, and alleviation of respiratory disturbance was noted in the patient who underwent lung transplantation.

As a surgical treatment for pericardial effusion, pericardiocentesis was performed,^396^, ^414^, ^415^, ^416^ and pericardial fenestration was performed when pericardial effusion could not be controlled by pericardiocentesis.^396^, ^416^ There was no mention of complications.

As sclerotherapy, pleurodesis was performed using OK‐432, talc and minocycline.^395^, ^397^, ^398^, ^399^, ^404^, ^408^, ^411^, ^416^, ^417^, ^418^ There were patients who responded markedly to sclerotherapy alone and sclerotherapy combined with surgical procedures such as pleural decortication or local radiotherapy. There was no mention of complications of sclerotherapy.

There have also been reports^399^, ^400^, ^402^, ^403^, ^404^, ^410^, ^411^, ^412^, ^415^, ^416^, ^418^, ^419^, ^420^ on local (e.g. lesion area, thoracic duct region) and thoracic radiotherapy for chylous pleural effusion and local lesions, and marked responses of chylous pleural effusion and responses of respiratory symptoms were noted, but other treatments were performed concomitantly in some patients. Radiation pneumonitis has been reported as a complication.^416^

Concerning nutritional therapy, fasting, high‐calorie infusion and MCT diet have been performed alone or in combinations, but few cases that showed alleviation of chylous pleural effusion were observed.^395^, ^396^, ^398^, ^399^, ^400^, ^403^, ^405^, ^408^, ^421^

For drug therapy against chylous pleural effusion, drugs including interferon (IFN)‐α, propranolol, anticancer agents (e.g. vincristine), bisphosphonate, octreotide, steroid, sirolimus and low‐molecular‐weight heparin were used. IFN‐α was used most frequently,^395^, ^396^, ^397^, ^398^, ^400^, ^401^, ^403^, ^415^, ^421^ and marked improvement in chylothorax was reported in five cases. Of these cases, IFN‐α was used with propranolol in one^395^ and with low‐molecular‐weight heparin and local radiotherapy (15 Gy) in one.^400^ As for complications of drug therapy using IFN‐α, there were reports of fever, nausea and headache,^421^ and thrombocytopenia and hepatic toxicity.^397^ There was no report of improvement in chylous pleural effusion by the use of steroid^395^, ^399^, ^403^, ^415^ or octreotide^395^, ^397^, ^398^, ^400^, ^403^, ^405^ alone. Concerning other drug therapies, only a few cases have been reported with no improvement in chylous pleural effusion. One case that showed regression of mediastinal invasion of GLA and alleviation of respiratory disturbance by sirolimus treatment has been reported,^414^ and hypertension was noted as a complication. In drug therapy for pericardial effusion, diuretics were used for conservative therapy.^400^

*Limitations*: Although cases that responded to various therapies have been reported, treatments are often performed in combinations, and the evaluation of the effectiveness of each treatment alone is difficult at this point.

## Summary

Treatments effective for GLA and GSD presenting with refractory chylous pleural effusion, pericardial effusion and respiratory disturbances were evaluated by a review of the published work, which was primarily case reports. Various treatments, such as surgery, sclerotherapy, radiotherapy, nutritional therapy and drug therapy, have been performed, but there was no study with a sufficient number of cases and a high level of evidence because of the rareness of the disease and diversity of symptoms. Although cases that responded to various treatments have been reported, treatments are frequently performed in combinations, and the evaluation of the effectiveness of individual therapies is difficult at present. Sirolimus (a mammalian target of rapamycin inhibitor) is considered promising as a drug for this disease, and some clinical trials are currently underway in Japan and abroad.

In clinical situations, these diseases are not recognized as indications of various drug therapies by the Japanese health insurance system, and the therapeutic effects of other treatments are also uncertain. Therefore, the above treatments cannot be recommended, but we propose that treatments “should be selected in consideration of complications and adverse effects according to individual symptoms”. It is necessary to evaluate the invasiveness, complications and adverse effects, and select the treatments judged to be appropriate for each case.

## CONCLUSION

These practice guidelines for vascular anomalies have been prepared as the EBM guidelines for the management of vascular anomalies.

## CONFLICT OF INTEREST

Conflict of interest of the guidelines preparation organization was managed by the Guidelines Executive Committee. The following corporations were disclosed by self‐declaration of the Guidelines Committee members in the 3‐year period before 1 April 2017: Japan Pharmaceuticals and Medical Devices Agency (PMDA), Mitsubishi Foundation, Rohto Pharmaceutical, Mitsubishi Tanabe Pharma and Shionogi.
